# Non-Furanic Humins-Based Non-Isocyanate Polyurethane (NIPU) Thermoset Wood Adhesives

**DOI:** 10.3390/polym13030372

**Published:** 2021-01-25

**Authors:** Xinyi Chen, Antonio Pizzi, Hisham Essawy, Emmanuel Fredon, Christine Gerardin, Nathanael Guigo, Nicolas Sbirrazzuoli

**Affiliations:** 1 LERMAB-ENSTIB, University of Lorraine, 27 rue Philippe Seguin, 88000 Epinal, France; xinyi.chen@univ-lorraine.fr (X.C.); hishamessawy@yahoo.com (H.E.); emmanuel.fredon@univ-lorraine.fr (E.F.); 2 Department of Polymers and Pigments, National Research Centre, Cairo 12622, Egypt; 3 LERMAB, Faculté des Sciences, University of Lorraine, Blvd. des Aiguillettes, 54000 Nancy, France; christine.gerardin@univ-lorraine.fr; 4 Department of Chemistry, University of the Cote d’Azur, 06103 Nice, France; Nathanael.GUIGO@univ-cotedazur.fr (N.G.); Nicolas.SBIRRAZZUOLI@univ-cotedazur.fr (N.S.)

**Keywords:** non-furanic humins, furanic humins, humic acid, fulvic acid, wood adhesives, wood panels, thermosetting adhesives, polyurethanes, NIPU

## Abstract

Predominantly non-furanic commercial humins were used to prepare humin-based non-isocyanate polyurethane (NIPU) resins for wood panel adhesives. Pure humin-based NIPU resins and tannin–humin NIPU resins were prepared, the latter to upgrade the humins’ performance. Species in the raw humins and species formed in the NIPU resins were identified by Matrix Assisted Laser Desorption Ionization Time of Flight (MALDI ToF) spectrometry and Fourier Transform Infrared (FTIR). Humins, fulvic acid and derivatives, humic acid and its fragments, some lignans present and furanic oligomers present formed NIPU linkages. Thermomechanical analysis (TMA) showed that as with other biomaterials-based NIPU resins, all these resins also showed two temperature peaks of curing, the first around 130 °C and the second around 220 °C. A decrease in the Modulus of Elasticity (MOE) between the two indicated that the first curing period corresponded to linear growth of the oligomers forming a physical entanglement network. This then disentangled, and the second corresponded to the formation of a chemical cross-linked network. This second peak was more evident for the tannin–humin NIPU resins. All the laboratory particleboard made and tested either bonded with pure humins or with tannin–humin NIPU adhesives satisfied well the internal bond strength requirements of the relevant standard for interior grade panels. The tannin–humin adhesives performed clearly better than the pure humins one.

## 1. Introduction

Recently, furanic humins derived from the acid treatment of pure fructose have been the focus of attention for the preparation of various foams and resins [1,2,3,4,5,6,7,8,9,10]. This interest was prompted by a Netherlands company having developed a novel process of industrialization to render commercially available this type of interesting material [11]. Unfortunately, up to today, such a process does not seem as yet to have been commercialized, simply because the production unit appears to be still being built.

However, mixed-furanic but predominantly non-furanic humins are abundantly produced by many factories around the world and on several continents. Such humins are a material obtained from humic substances, particularly rich in humic and fulvic acids. The organic components of soil can be subdivided into fractions that are soluble, largely humic acids, and insoluble, the humins.

Humins make up about 50% of the organic matter in soil. Humins are also produced during the dehydration of sugars, as occurs during the conversion of lignocellulosic biomass to smaller, higher-value organic compounds such as 5-hydroxymethylfurfural (HMF). These humins can be in the form of either viscous liquids or solids depending on the process conditions used. Such humins have a polymeric furanic-type structure, with hydroxyl, aldehyde and ketone functionalities. However, their structure is dependent on feedstock type (e.g., xylose or glucose) or concentration, reaction time, temperature, catalysts and many other parameters involved in the process [12]. They are used in agriculture to enhance fertilizers’ effect and absorption by increasing soil fertility, as foliar fertilizers, in the pharmaceutical-cosmetic field [13], in the preparation of catalytic materials [14] and in material applications. However, depending on their origin they appear to predominantly contain humic acids, fulvic acid and also a minor but still noticeable proportion of furanic materials.

Polyurethanes are ubiquitous and very versatile products used today for a great variety of material applications. They are industrially prepared by the reaction of polyols with isocyanates. Great strides have been successfully made to prepare them from biosourced polyols. However, isocyanates are the key material to produce them, but they present the problem to be classed as toxic. It is for such a reason that chemical synthesis routes to prepare non-isocyanate polyurethanes have been developed [15,16,17,18,19,20,21,22,23]. Polyurethanes are used as wood adhesives in structural glulam and finger-jointing. Polymeric diphenylmethane diisocyanate (p-MDI) is also used for wood panel adhesives. The toxicity of p-MDI has again focused research on its substitution by preparing polyurethanes without isocyanates (NIPU).

Several approaches are used to prepare non-isocyanate polyurethanes, namely by reacting hydroxyl group-carrying materials with one or two cycles organic carbonates or CO_2_ and diamines [15,16,17,18,19,20,21,22,23,24]. All these are using oil-derived synthetic-based materials to prepare NIPUs. More recently, successful attempts to produce NIPUs from natural materials rich in hydroxyl groups have been reported [25,26,27,28,29,30,31,32,33,34,35]. These have the additional characteristic to have simplified the procedure by using a cheaper aliphatic carbonate by eliminating the reaction of preparing cyclic carbonates [25,26,27,28,29,30,31,32,33,34,35]. Among these, glucose-and sucrose-based NIPUs prepared with dimethyl carbonate and hexamethylene diamine were used to bond wood joint and particleboard with encouraging results [32,33].

Thus, non-isocyanate polyurethanes are an interesting route to pursue, considering mixed predominantly non-furanic humins are rich in a number of hydroxyl groups and amino groups. The work described here is then aimed at preparing an NIPU thermosetting wood panel adhesive based on predominantly non-furanic humins and presenting good bonding characteristics.

## 2. Materials and Methods 

### 2.1. Materials

Gerhumin^®^ (a registered predominantly non-furanic humins solution) was supplied by L.Gobbi S.r.L. (Campo Ligure, Italy). Gerhumin^®^ is extracted in Germany by E. Gerlach GmbH (Lübbecke, Germany). Gerhumin^®^ solution contains 21% solids, 80% of which are organic materials, and 61% of the total solids are humic substances, with 0.7% organic nitrogen on dry material and C/N ratio of 57.1, and pH 9–10. Commercial Mimosa bark condensed tannin extract was supplied by Silva Chimica (St. Michele Mondovì, Italy). Hexamethylene diamine 98%, and dimethyl carbonate (DMC) were bought from Sigma-Aldrich (Saint Louis, France). 

### 2.2. Preparation of Humins-NIPU Adhesives

Four resins were prepared using Gerhumin^®^. One was prepared with the humins solution alone. Three of them were prepared using Gerhumin^®^ in combination with mimosa bark tannin extract, this latter used to increase the bonding strength of the humin-based adhesives.

Resin A,
  Step 1: 20 g tannin + 15 g Gerhumin^®^ + 5 g H_2_O +13.5 g DMC, at 60 °C for 1.5 h;
  Step 2: 38.8 g of diamine, at 90 °C for 2 h.
  Resin characteristics: viscosity at 23 °C 1620–1650 mPa.s; solids content 54.5%.
Resin B,
  Step 1: 40 g Gerhumin^®^ + 13.5 g DMC, at 60 °C for 1.5 h;
  Step 2: 38.8 g of diamine, at 90 °C for 2 h.
  After the end of the reaction, to obtain the final solid content of 45%, rotary evaporation under reduced pressure was used.
  Resin characteristics: viscosity at 23 °C 520–540 mPa.s; solids content 45%.
Resin C,
  Step 1: 20 g tannin + 16.67 g H_2_O+ 13.5 g DMC, at 60 °C for 1.5 h;
  Step 2: 15 g Gerhumin^®^, 90 °C for 1 h;
  Step 3: 38.8 g of diamine, 90 °C for 1 h.
  Resin characteristics: viscosity at 23 °C 1050–1120 mPa.s; solids content 52.3%.
Resin D,
  Step 1: 20 g tannin + 16.67 g H_2_O + 13.5 g DMC, at 60 °C for 1.5 h;
  Step 2: 38.8 g of diamine + 15 g Gerhumin^®^, at 90 °C for 2 h.
  Resin characteristics: viscosity at 23 °C 1230–1310 mPa.s; solids content 51.7%.

### 2.3. ATR-FT-MIR Analysis

All of the samples were analyzed with a Perkin-Elmer Frontier ATR-FT-MIR provided by an ATR Miracle diamond crystal. The powder and liquid samples were laid on the diamond eye (1.8 mm) of the ATR equipment and the contact for the sample was ensured by tightly screwing the clamp device. Each extract was scanned registering the spectrum with 32 scans with a resolution of 4 cm^−1^ in the wave number range between 600 and 4000 cm^−1^.

### 2.4. Matrix-Assisted Laser Desorption Ionization Time-of-Flight (MALDI-ToF) Spectrometry Analysis

The original Gerhumin solution and the four resins prepared were examined by MALDI ToF spectrometry. The samples for analysis were prepared by first dissolving 7.5 mg of sample powder in 1 mL of a 50:50 v/v acetone/water solution. Then 10 mg of this solution was added to 10 µL of a 2,5-dihydroxy benzoic acid (DHB) matrix. The locations dedicated to the samples on the analysis plaque were first covered with 2 µL of a NaCl solution 0.1 M in 2:1 v/v methanol/water, and pre-dried. Then 1.5 µL of the sample solution was placed on its dedicated location and the plaque was dried again. Red phosphorous was to standardize the MALDI equipment. MALDI-ToF spectra were obtained using an Axima-Performance mass spectrometer from Shimadzu Biotech (Kratos Analytical Shimadzu Europe Ltd., Manchester, UK) using a linear polarity-positive tuning mode. The measurements were carried out making 1000 profiles. The spectra were accurate to ±1 Da.

### 2.5. Thermomechanical Analysis (TMA)

The resins were tested by thermomechanical analysis. Matched wood plies were used to minimize the variation due to the wood substrate used. The samples were prepared by applying each adhesive between two beech wood plies, with dimensions of 21 × 6 × 1.1 mm. These beech-resin-beech sandwiches were tested in non-isothermal mode between 25 and 250 °C at a heating rate of 10 °C/minute with Mettler Toledo 40 TMA equipment (Mettler Toledo, Zurich, Switzerland). They were tested in three-point bending on a span of 18 mm exercising a force cycle of 0.1/0.5 N on the specimens, with each force cycle of 12 s (6 s/6 s). The classical mechanics relationship between force and deflection can be expressed as

E = [L^3^/(4bh^3^)][F/(f_wood_ − f_adhesive_)]

where L = the length of the sample; b = its width; h = its thickness; F = the force applied; f_wood_ is the deflection of the wood and f_adhesive_ the deflection of the wood adhesive sandwich under test. This equation allows the calculation of the Young’s modulus E (modulus of elasticity, MOE) for each case tested. Such a measuring system has been introduced and is used to follow the progressive hardening of the adhesive with the increase of temperature and to indicate comparatively if an adhesive system is faster or slower hardening and if it gives stronger joints than another one.

### 2.6. Wood Particleboard Preparation and Testing

Duplicate one-layer particleboard panels of 350 × 350 × 14 mm dimensions were prepared by adding 10% of the resin solids mixture on dry wood particles for a percentage wood moisture content of the resinated particles of 13% and pressed at a maximum pressure of 28 kg/cm^2^, followed by a pressure-decreasing pressing cycle at 220 °C for a 10 min pressing cycle. The target density of the panels was 720 kg/m^3^. The panels, after light surface sanding, were tested for dry internal bond (IB) strength (EN 312) [36].

## 3. Results and Discussion

The dry internal bond (IB) strength results of the laboratory one-layer wood particleboard panels are shown in Table 1. The results indicate that all four types of NIPU resins satisfied the requirement of the relevant European norm [36] for interior grade particleboard panels. It can be noted that the pure humins NIPU resin presented the lowest IB strength value, although this was still well above the requirements of the standards. The tannin–humin NIPU presented better IB strength although these values were rather different for each resin due to the differences in their preparation procedure. Resin A was prepared by premixing tannin and humin and carbonating their mixture with DMC, then the carbonated mixture was reacted with the diamine. 

Resin C and D were prepared using the same material proportions but with a different procedure. Thus, in resin C the tannin alone was first carbonated by reaction with DMC, then reacted with the humins for 1 h, and only after this was the mixture reacted with hexamethylene diamine for 1 h.

In resin D, the tannin alone was first carbonated with DMC, then reacted with the premixed humins and diamine.

The difference in preparation procedure does account for the different bonding performance of the A, C and D adhesives, although these differences are not too big. Thus, in resin A, both tannin and humins were carbonated simultaneously and almost equally, ensuring a greater variety of urethane-like linkages in the resin. 

In resin C, the tannin was instead first and predominantly carbonated, with the humins reacting both with the pre-carbonated tannin but also with the DMC still unreacted. This means that in resin C one could expect urethane linkages mainly, but not only, involving the tannin, but with the humin-linked urethanes being possibly in the minority.

In resin D, the pre-carbonated tannin was instead reacted with the humins + diamine premix, most likely ensuring a more equitable partition of urethane linkages on both tannin and humins. 

The IB strength results confirm that addition of tannin to yield mixed tannin–humin adhesive resins yield better results than the humins alone. The IB strength results also confirm these differences with resin A and resin D yielding a better IB strength due to the better partition of urethane linkage between tannin and humins, while in resin C a slightly larger part of the humins were either unreacted or not linked. 

**Figure 1:**
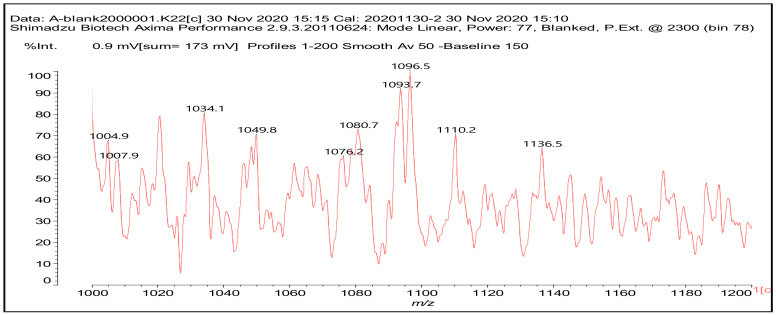
An example of matrix-assisted laser desorption ionization time-of-flight (MALDI ToF) spectrum of NIPU resin (D) in the range 1000–1200 Da.

It is of interest to determine which species were formed for the different resins and what for each of them were the predominant ones. Thus, the structures present in the mix of predominantly non-furanic humins were determined by MALDI ToF (Figure 1). The mix was composed mainly of fulvic acid (I), fulvic acid derivatives (II) and (III), several humic acid fragments (IV) and several other non-furanic structures, and also of some furanic oligomers. Some mono- and di-lignans were also present. The list of the structures present and the relevant MALDI ToF spectra are shown in the Supplementary Materials (Table S1, Figure S1a–g).

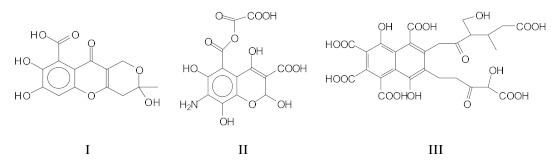

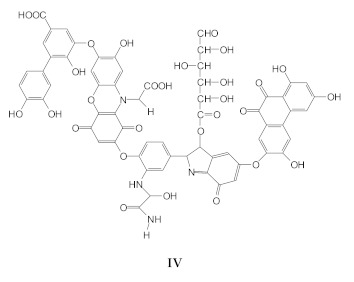





The reaction of the four different formulations of thermosetting NIPU resins confirms the co-reaction of the different components. Thus, for the NIPU resin prepared from non-furanic humins the oligomers formed were urethane-bonded species that had formed by reaction of fulvic acid and derivatives with DMC and diamine, and also furanic species reacted to form NIPUs by reaction with DMC and diamine. These are shown by the following species assigned to the MALDI ToF spectrum peaks at 364 Da (V) furanic, 452 Da (VI) fulvic, non-furanic, with two possible structures at 512 Da (VII) fulvic, non-furanic.

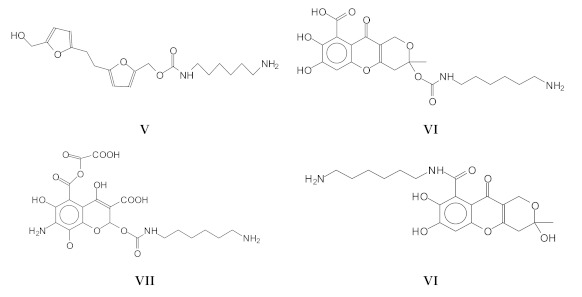



The two possible (VI) structures indicate that the urethane bond can form either on the alcoholic –OH of the fulvic acid structure, or alternatively could form an amide by reaction on the acid moiety of fulvic acid.

Characteristic dimers and higher oligomers were also found, such as at 796 Da (VIII) non-furanic and with different species linked by the urethane linkages, such as at 847 Da (IX) non- furanics.

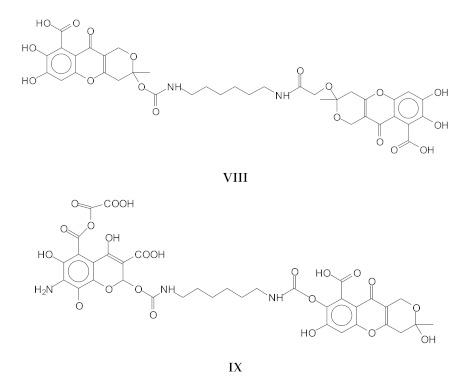



Fulvic acid-derived urethanes linked to lignans present in the predominantly non-furanic humins mix were also identified, such as at 1193 Da (X).

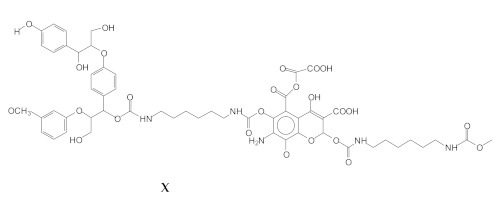



Equally present were some more rare cyclic structures obtained from fulvic acid derivatives where this was possible, such as for the structure at 828 Da (XI).

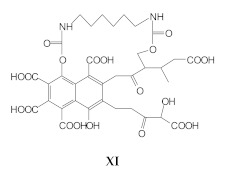



For the other adhesive resins where mimosa tannin was used as a reaction support for the non-furanic humins mix to prepare the resins, structures involving mixed urethane oligomers involving all the different types of species were identified. Thus urethane linkages between two flavonoids did occur, such as at 732 Da (XII), and between a flavonoid and fulvic acid at 781 Da (XIII), and between flavonoids and humic acid fragments at 1096 Da (XIV).

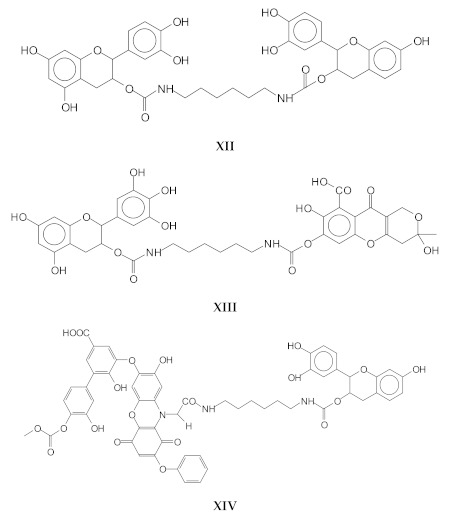



Structures of the type shown as (VI) and (XI) were also present. The complete list of the compounds identified for the resins supported by the tannin are shown in the Supplementary Materials (Tables S2 and S3, Figures S2a–d and S3a–f).

It is of equal interest which of these species were in the majority in resins A, C and D to confirm if the hypothesis of their different IB strength depending on the more or less fair distribution of urethane linkages is correct or not (Table S4, Supplementary Materials). From Table S4, where are indicated the more intense significant MALDI spectra peaks for each resin, it is evident that in the case of resins A, C and D the main species contributing to the positive IB strength results obtained for the wood particleboards bonded with these resins were species, such as species XIII and XIV in which tannin and humins form mixed urethane-linked species and networks.

**Figure 2:**
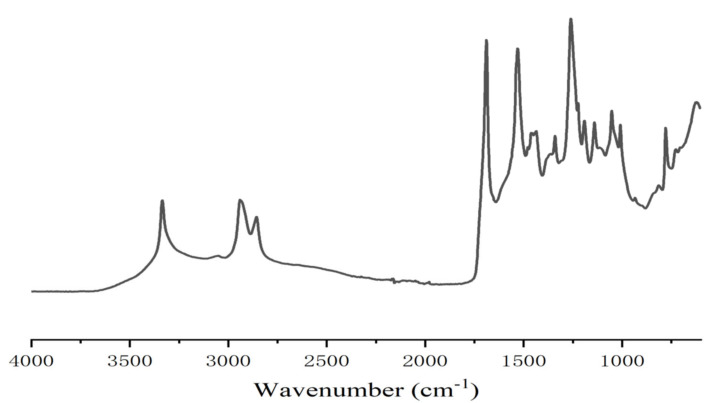
FTIR spectrum of tannin–humin NIPU resin A.

**Figure 3:**
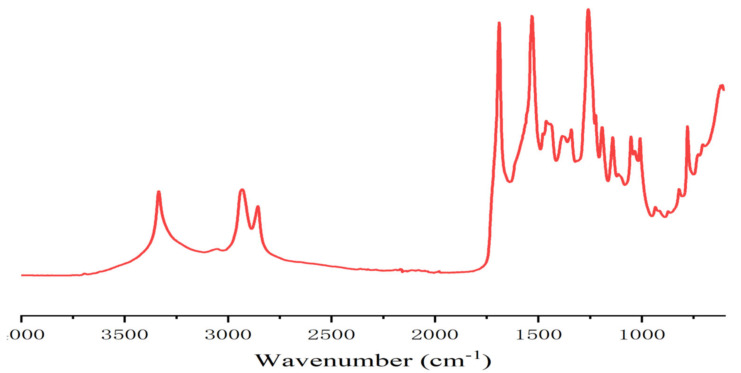
FTIR spectrum of pure humin NIPU resin B.

**Figure 4:**
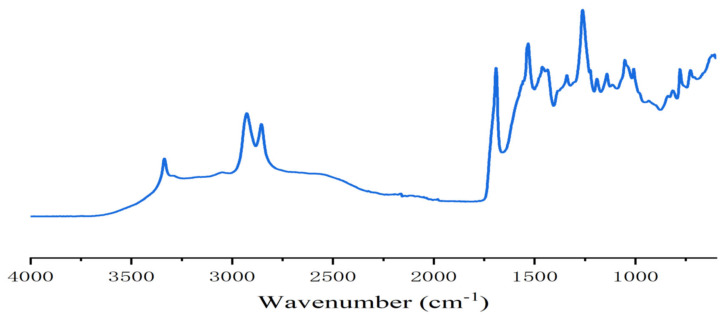
FTIR spectrum of tannin–humin NIPU resin D.

**Figure 5:**
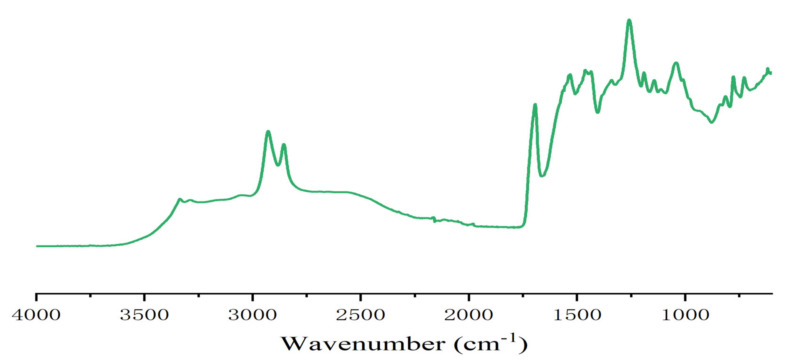
FTIR spectrum of tannin–humin NIPU resin C.

In the case of resins A and B (Figure 2 and Figure 3) the FTIR spectra contribute to interpreting what occurred. The presence of urethane linkages was confirmed by the presence of the asymmetric stretching of the urethane linkages represented by the sharp peak at 3400 cm^−1^ coupled with the peaks at 1711–1740 cm^−1^ of the urethane C=O peak, and the peaks at 1538, 1250 and 1062 cm^−1^. These four latter peaks are a sum of the urethane linkage and of the carbonate stretching being superimposed, while the peak at 1711–1740 cm^−1^ is also contributed to by the C=O of the carboxylic acid functions present, this being confirmed by the presence of the very small but clear peak at 923 cm^−1^ in resin B assigned to the few carboxylic acids present in the humins. This can be seen from the difference between the spectra of resin A and resin D (where in the second the 3400 cm^−1^ peak was also much lower as the carbonate contribution had disappeared) and the small peak of the carbonate at 1230 cm^−1^. The other peaks present were the two sharp peaks at 2970 and 2860 cm^−1^ assigned to asymmetric and symmetric stretching of alkane chains, and the peaks characteristics of ether groups, present in both the humins and the tannin at 1240 (shoulder), 1029 and 832 cm^−1^. In the case of the FTIR spectrum of resin B, there was also a small but clear peak at 923 cm^−1^ assigned to the few carboxylic acids present in the humins. The profile of resin C was slightly different from the profile of resin A (and D) with the peaks indicating the formation of urethanes, in particular the peak at 3400 cm^−1^ being of much lower intensity (Figure 2, Figure 4 and Figure 5). In fact, the procedure of preparation of resin C at lower temperature, coupled with the order of addition of the reagents, suggests that the proportion of urethane linkages in this resin was lower than in resins A and D. This is supported also by the lower IB strength of the particleboards bonded with it in Table 1.

**Figure 6:**
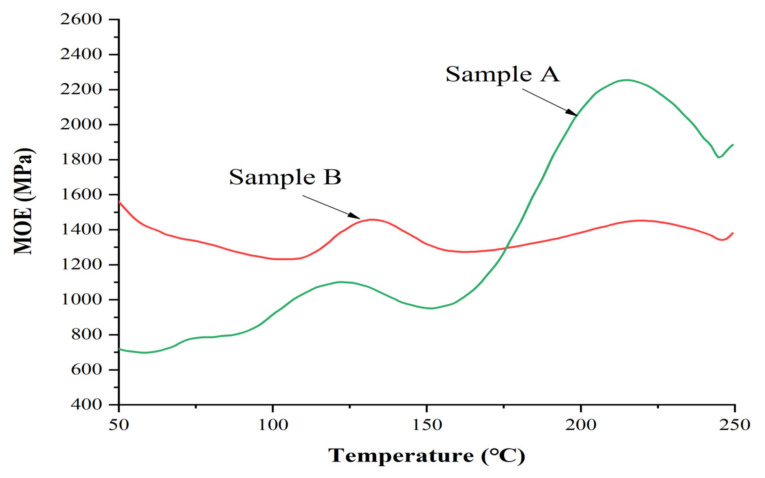
Thermomechanical analysis (TMA) traces for resins A and B.

It is of interest to try to understand why resin B, which was a humins-only-based NIPU resin, gave IB strength values that, while passing the requirements of relevant standards, were much lower than the tannin–humin NIPU resins A, C and D. The reasons could be several, such as a higher energy of activation of curing for resin B, a lower reactivity of the resin or a lower level of cross-linking due to a lower proportion of reactive sites on the humins themselves. For this, curing of the resins in a thermomechanical apparatus showed that the increase of the modulus of elasticity (MOE) as a function of the increase in temperature was very different for resins B and A (which had a similar curve to that of resins C and D) (Figure 6). In Figure 6 both resins A and B show two peaks of the MOE value, but while the first peak is of similar intensity, the second one has a much higher MOE value for resin A. Moreover, the first peak has its maximum at 120 °C for resin A and at 135 °C for resin B, while the second peak occurs at the equivalent temperature of 220 °C for both resins. 

The two peaks were due to the formation of hardened networks of different nature. The higher temperature of the first peak for resin B indicates that this resins presented possibly a higher energy of activation of curing, possibly coupled with the lower solids content and its lower viscosity. The reason of the much higher modulus of elasticity (MOE) value of the second peak for resin A was due to a far better chemical cross-linking due to the contribution of the very reactive tannin to the network. The differences in the intensity of the second peak for resins A and B cannot be ascribed to differences of the wood substrate, as matched wood samples of even grain from the same piece of veneer were used. Thus, the inference is that the pure humins resin B had a level of ultimate cross-linking much lower due to a lower proportion of reactive sites on the humins themselves, as evidenced looking at the respective structures involved.

The lower temperature peak indicates the formation of an unstable network that is degraded and transformed if the temperature is increased, causing a marked internal rearrangement. The second peak at higher temperature indicates that curing of the NIPU resin occurred at a high temperature, as already reported in previous literature for similar types of adhesives [32,33]. The increase of Young’s modulus has been shown to correlate with the bonding strength of the adhesive [37,38,39,40,41,42].

It is interesting to speculate as to what was the cause of the marked decrease between the two modulus of elasticity (MOE) peaks in Figure 6. While it could be an unstable structure, a more likely reason could derive from the increase in length of linear oligomers progressively forming a physically entangled network, rather than a chemically cross-linked one. As the temperature increased further, the Brownian movements would make the viscosity of these physically entangled chains markedly decrease and extensively disentangle. The modulus of elasticity (MOE) then started again to increase at the much higher temperature of the second peak once chemical cross-linking finally occurred. 

Before the TMA curve of the B resin showed an increase in the modulus value, the overall trend of the modulus value decreased between 50 and 110 °C. This was possibly due to the lower solids content, lower viscosity and higher water allowing the decrease of viscosity due to the increase of temperature to be the effect that predominates on the slow oligomers linear growth. Resin A did not show this trend, its initial viscosity allowing the linear growth of the oligomers to be detected by a slow initial increase of the modulus of elasticity (MOE). Conversely, the adhesive penetrated into the wood, softening or degrading it during heating, thus reducing its strength. It is evident from the TMA traces that the curing temperature leading to chemical cross-linking was high, starting at 150−160 °C. This means that the hardening temperature to be used for such adhesives is relatively higher than what is common today for equivalent panels bonded with traditional adhesives. After 200 °C, degradation of the wood substrate is known to start [37,40,41,42], thus for the second peak one needs to consider that wood degradation had already started to occur at the same time, and thus the hardening of the resin was better than what appears from the TMA curves for both resins. The decrease of the curve after the second peak was due to the continuing degradation of the wood substrate.

## 4. Conclusions

Non-isocyanate polyurethane (NIPU) resins were prepared using commercial humins predominantly composed of fulvic acid and its derivatives, and of humic acid, but also presenting some polyfuranic and lignan structures in their mix. The NIPU resins were prepared by using just the humins or by using a mimosa-condensed tannin–humin mix. FTIR and MALDI ToF spectrometry allowed the identification of the original constituents mix in the humins as well as the NIPU oligomers formed in the reactions used. It appeared that all the different constituents in the humins reacted to form NIPU oligomers. Thus, fulvic acid and its derivatives, humic acid and its fragments, and the furanic and lignan structures present all reacted to form NIPU constituents and contributed to the formation of NIPU resins. Interestingly, humins constituents NIPU oligomers, tannin NIPU oligomers and even several mixed NIPU species derived from the co-reaction of condensed tannin units and humins constituents were also formed. These latter ones showed that in the case of the tannin–humin NIPU both the two biomaterials formed a unitary, all-linked network. TMA was used to assess the resins’ curing behavior and showed that two temperatures of curing appeared to occur, the first at around 130°C and the second around 220 °C. The first appeared to indicate the linear growth of oligomers leading to a physically entangled network which then disentangled and proceeded at the higher temperature to form a chemically cross-linked network. The final cross-linked network appeared to be markedly less cross-linked for the pure humins NIPU resin than for the tannin–humin ones. This was confirmed by the internal bond (IB) strength values of the laboratory wood particleboard bonded with the two types of resin, which was lower for the pure humins NIPU resin than for the tannin–humin ones. Nonetheless, the IB strength values of all the boards bonded with the two types of NIPU resins satisfied the requirements of the European norm for interior grade particleboards.

## Figures and Tables

**Table 1:** Results of particleboard tests with the experimental non-furanic-based non-isocyanate polyurethane (NIPU) wood adhesives.

Samples	Density (g/cm^3^)	Internal Bond (MPa)	Bending Strength (MPa)	Elastic Modulus (MPa)
A-220 °C	0.77 ± 0.03	0.70 ± 0.08	14.1 ± 1.4	6700.8 ± 523.5
B-220 °C	0.73 ± 0.02	0.44 ± 0.04	6.4 ± 0.4	968.6 ± 34.2
C-220 °C	0.73 ± 0.01	0.65 ± 0.07	12.9 ± 2.0	4081.5 ± 56.0
D-220 °C	0.75 ± 0.03	0.74 ± 0.01	14.4 ± 2.7	4805.2 ± 80.7
EN requirements		>0.35		

## Supplementary Materials

The following are available online at https://www.mdpi.com/2073-4360/13/3/372/s1, Table S1. Assignments of oligomer structures present in Gerhumin predominantly non-furanic humins; Table S2. MALDI ToF assignment of oligomers present in tannin–Gerhumin NIPU resin A; Table S3. MALDI ToF assignment of oligomers present in pure Gerhumin NIPU resin B; Table S4. Relative intensity of MALDI peaks for non-furanic humins and tannin–humin NIPU resins; Figure S1a–g: MALDI ToF spectra details of different Da intervals of raw Gerhumins, corresponding to the structures assigned in Table S1; Figure S2a–d: MALDI ToF spectra details of different Da intervals of tannin–Gerhumin NIPU resin A, corresponding to the structures assigned in Table S2; Figure S3a–f: MALDI ToF spectra details of different Da intervals of pure Gerhumin NIPU resin B, corresponding to the structures assigned in Table S3.
