# Supplementary material for: Non-Furanic Humins-Based Non-Isocyanate Polyurethane (NIPU) Thermoset Wood Adhesives
Source: Polymers (Basel). 2021 Jan 25;13(3):372. doi: 10.3390/polym13030372 (PMC7865859; doi:10.3390/polym13030372)
Supplement: Supplementary file 1 [file polymers-13-00372-s001.pdf]

SUPPLEMENTARY MATERIAL

NON-ISOCYANATE POLYURETHANES (NIPU) THERMOSETTING WOOD ADHESIVES BASED ON PREDOMINANTLY NON-FURANIC HUMINS

X.Chen, A.Pizzi*, H.Essawy, E.Fredon, C.Gerardin, N.Guigo, N.Sbirrazzuoli

Table S1. Assignements of oligomer structures present in Gerhumin predominantly non-furanic humins

---

FURANIC PART OF GERHUMINS

139 Da both theoretical and experimental, no Na+

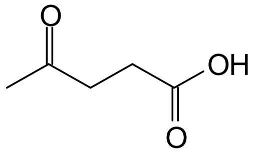

139 Da (calculated 140) no Na+

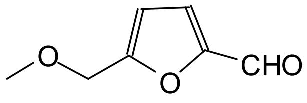

159 Da (calculated 158 Da) no Na°

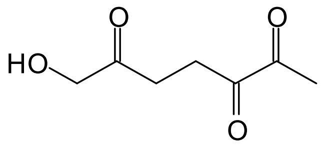

159 Da (calculated 156 Da) no Na+

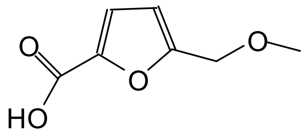

177-179 Da (Calculated 178 Da) with Na+

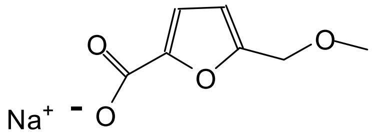

202-203 Da (Calc 203) with Na+

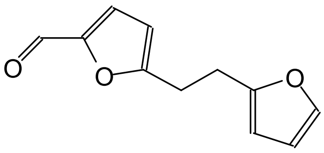

203 Da (Calc 203 Da) with Na+) (1d & e, thesis St Gregorio, Fig.2.2.3)

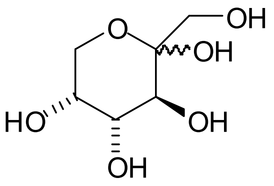

244-245 Da = with Na+

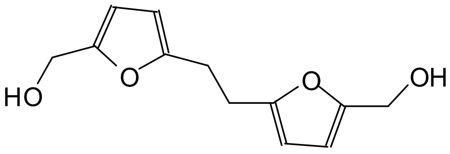

257 Da (calc 252 Da)

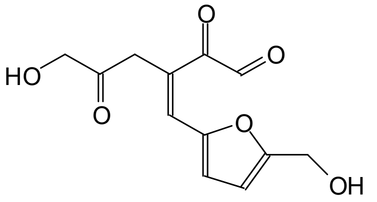

257 Da (Calc 254 Da)

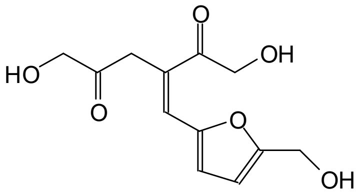

284 Da no Na+ (150°C and 180° oxidized spectra)

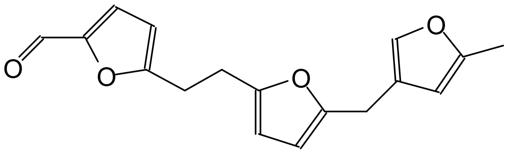

391 Da (Calculated 389 Da) with Na+, (180°C not oxidieed)

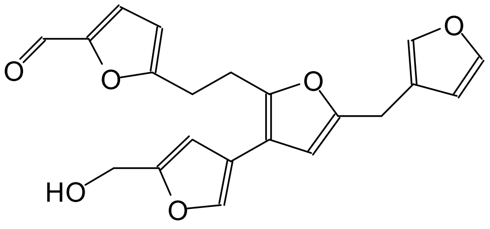

392 Da (Calculated 396 Da) (180°C not oxidized) (Thesis St.Gregorio, Fig. 2.2.2)

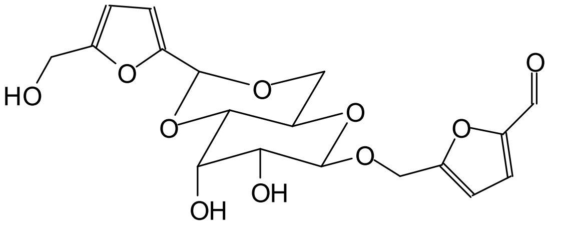

428.5 Da , without Na+

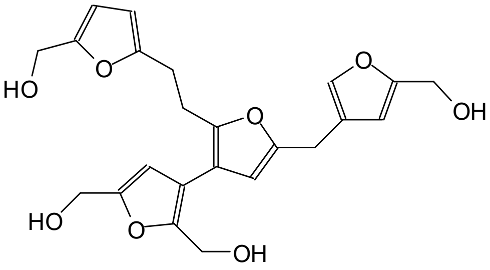

636 Da (Calculated 637 Da) with Na+, (180°C not oxidized spectrum)

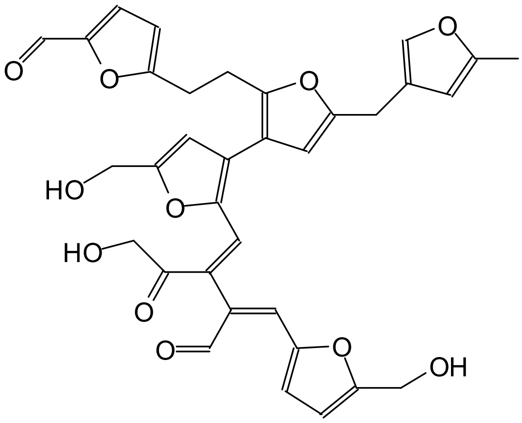

660-661 Da without Na+ and **685 Da** with Na+ could also be the species

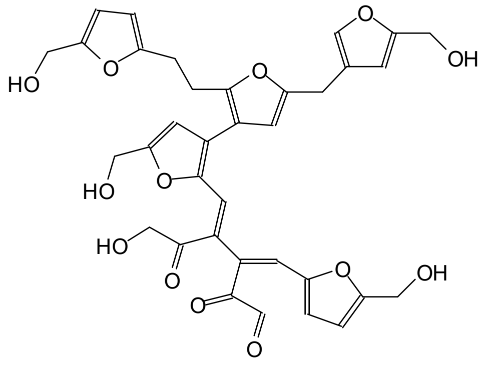

935 Da (Calculated 932 Da) without Na+ (180°C Oxidized, 150°C

950-952-954 Da (Calculated 955 Da) with Na+ (180°C oxidized and not oxidized)

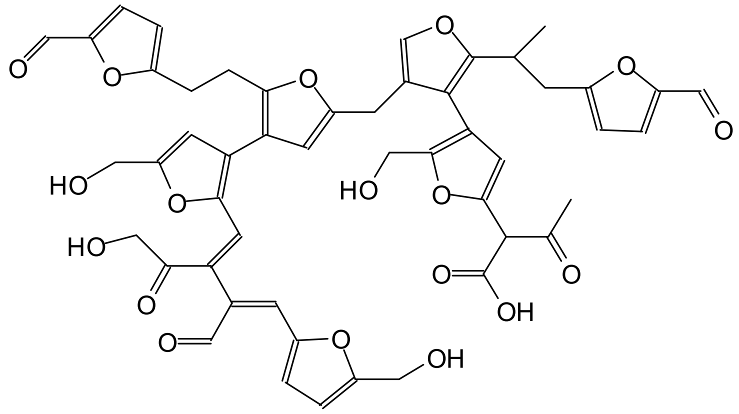

1010 Da (Calculated 1012 Da) no Na+ (180°C not oxidized)

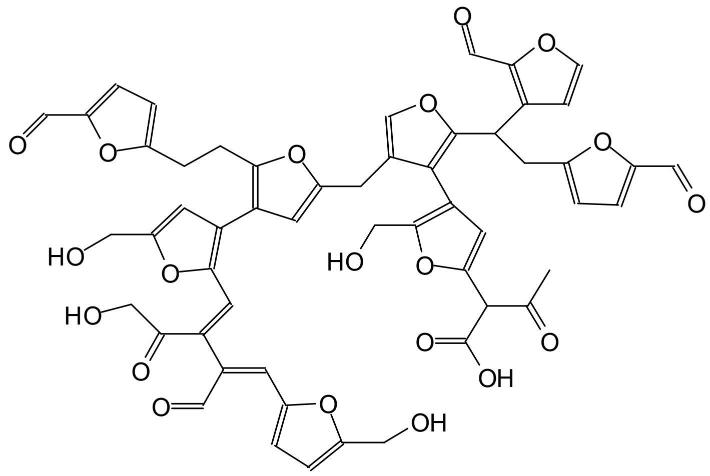

NON-FURANIC SECTION OF GERHUMINS

307 Da = fulvic acid structure, no Na+, calculated 308 Da

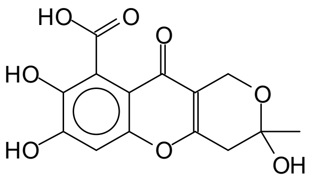

311 Da = with Na+, Calculated 311 Da. **A lignin monomer**

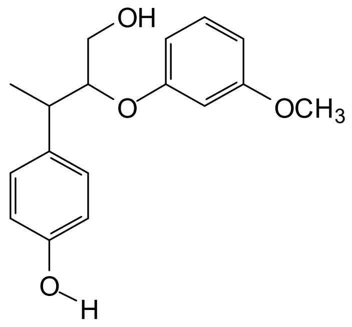

479 Da = with Na+, calculated 477 Da **a Lignan**

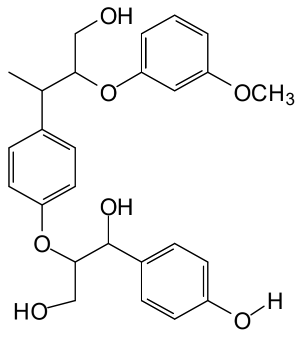

MW = 308 there is a 307 Da no Na+ and a 329/333 Da with Na+

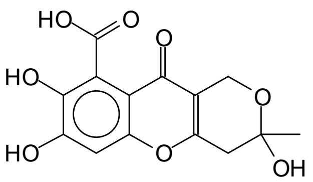

361 Da = 393 Da – 2xOH

376 Da = 393 - 1xOH

393 Da = without Na+, but with just single O, with Na+

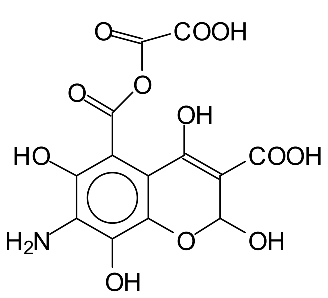

409 Da = 393 + 1x-OH and **425 Da** = 409+ 1x-OH

638 Da =no Na+

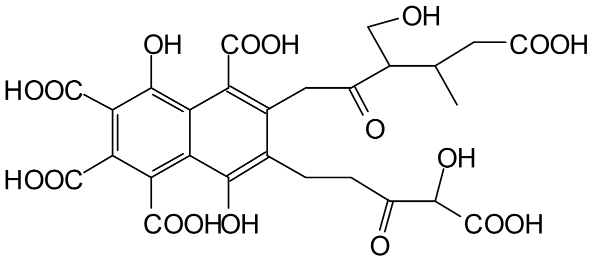

570 Da = with Na+ (same structure at **549-551 Da** [calculated 547 Da] without Na+) and **592 Da** with 2xNa+

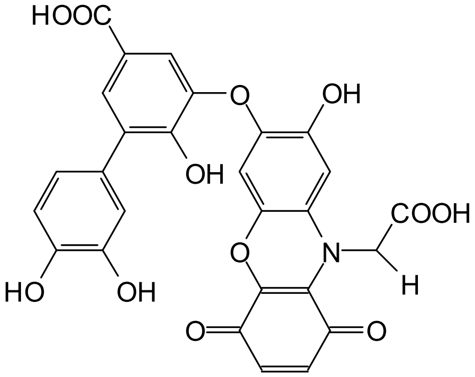

602 Da = 570 Da + 2xOH

638.4 Da = no Na+, calculated 639

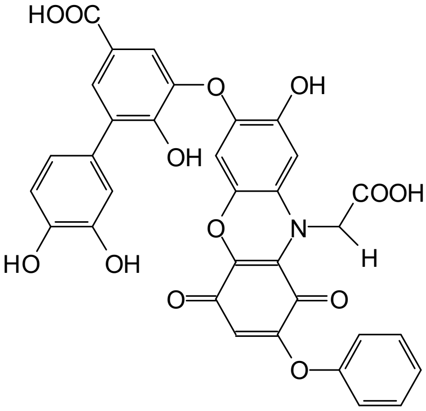

MW= 638 there is a clear **638 Da** no Na+ and at **659-661** Da with Na+ and **685 Da** with 2xNa+

701 Da = 685 Da + 1x-OH

660-661 Da without Na+ and **685 Da** with Na+ could also be the species

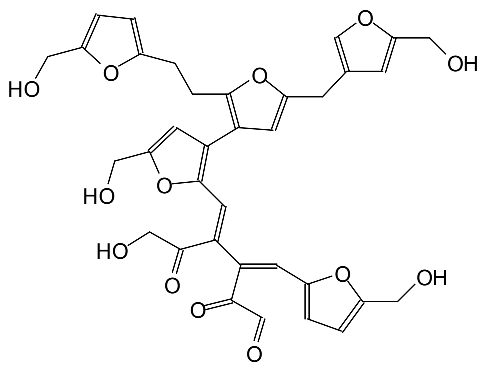

699 Da - 703 Da = with Na+, Calculated 703 Da, **a lignan trimer**

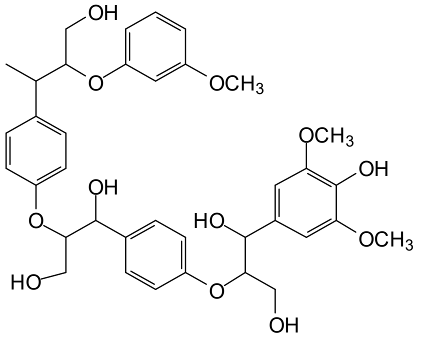

714 Da

747 Da = with Na+, calculated 748 Da

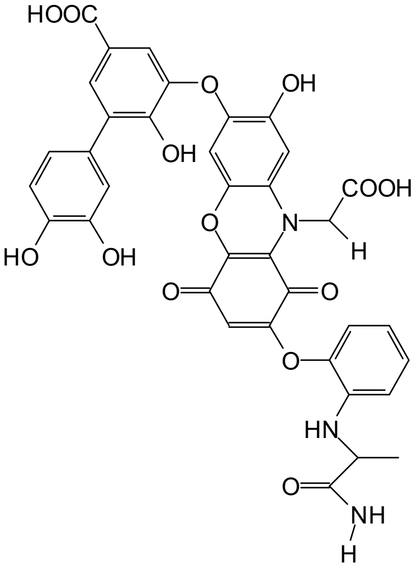

855 Da = without Na+, calculated 856 Da, and **878 Da** with Na+ and **901 Da** with 2x Na+ and **924 Da** with 4x Na+

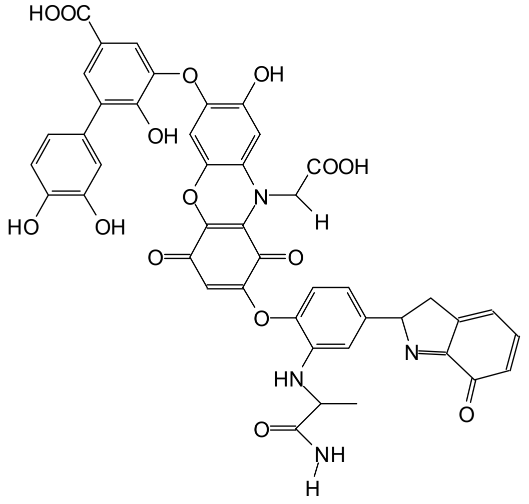

1051 Da without Na+ and **1097Da** +2xNa+

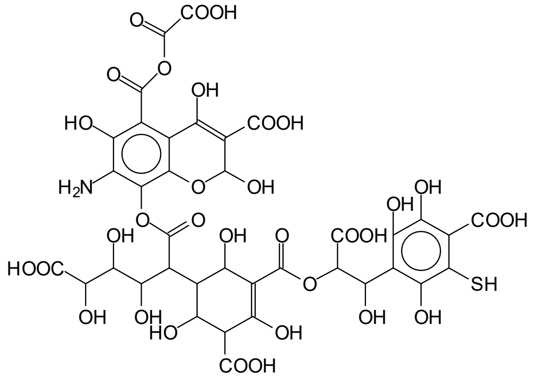

1057 Da = with Na+, calculated 1057 Da. But **1079 Da** with 2x Na+

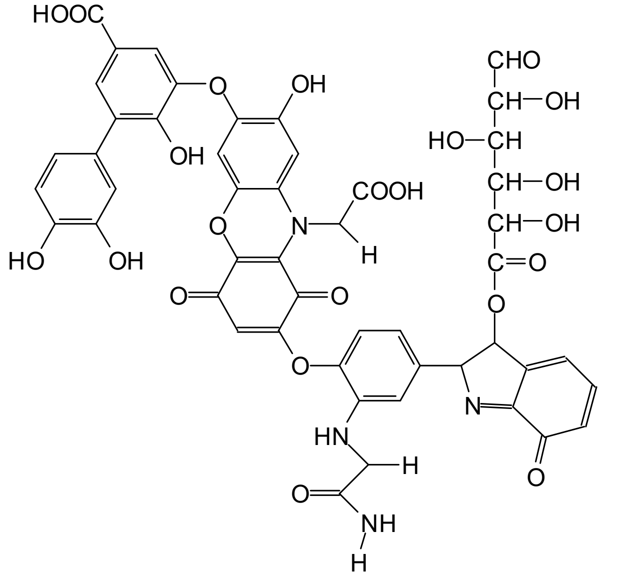

1166 Da = with Na+, Calculated **1165 Da**

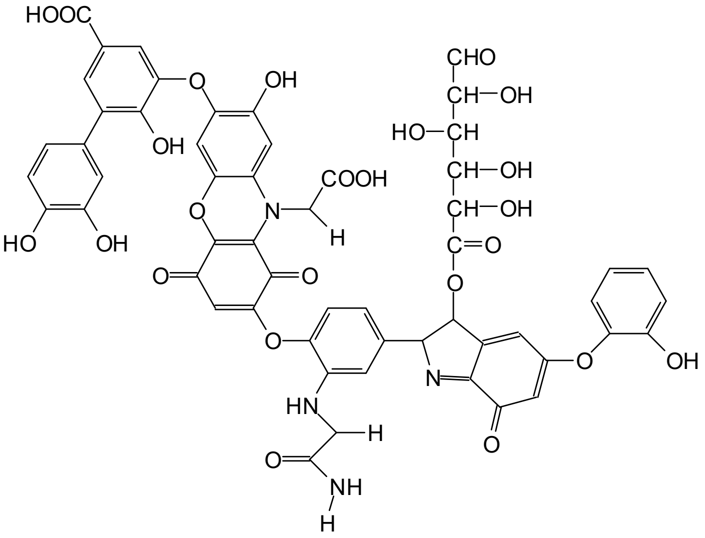

1175 Da = without Na+ (calculated 1175 Da), and **1200 Da** (calculatred 1198 Da) with Na+

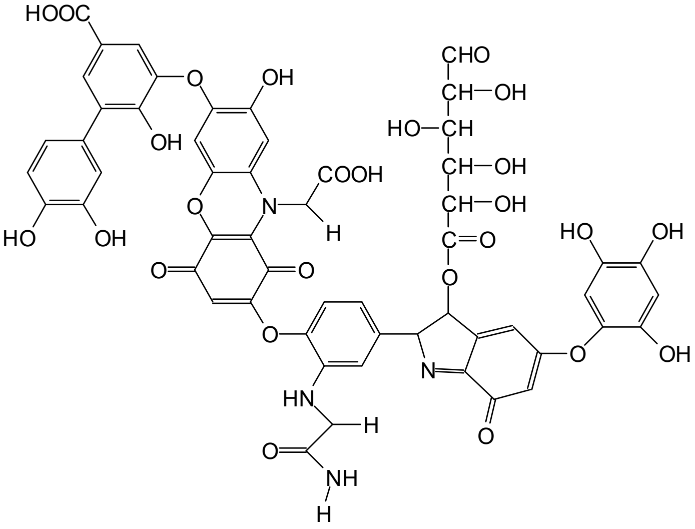

1344 Da =with Na+, calculated 1344 Da

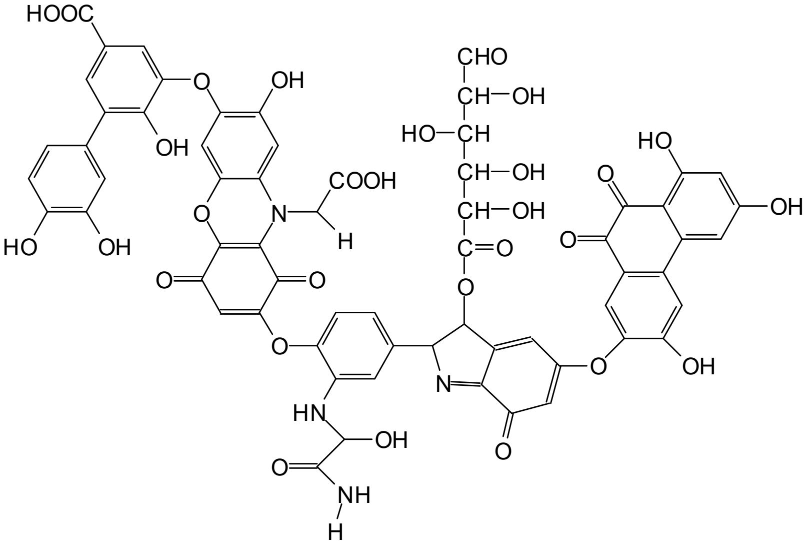

The ABOVE INDICATES THAT THE PROPOSED STRUCTURE HEREUNDER IS PRESENT **Tag Archive : fulvic acid** https://www.humicacid.org/tag/fulvic-acid/

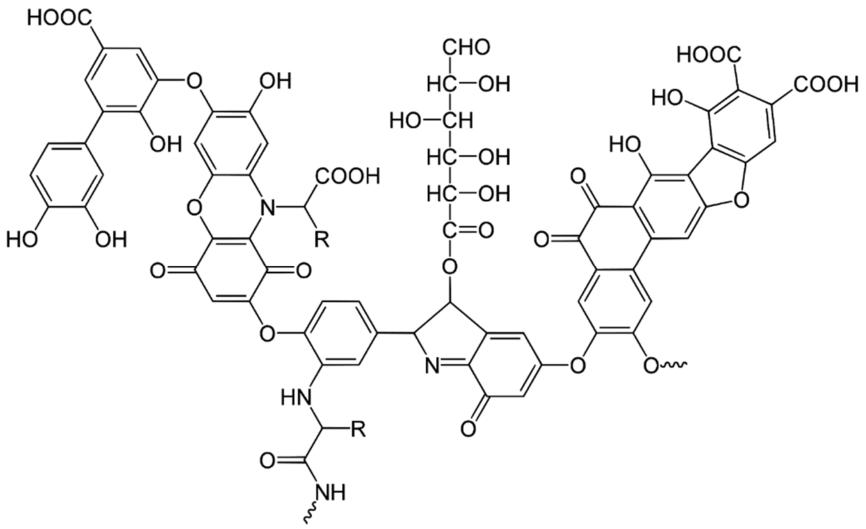

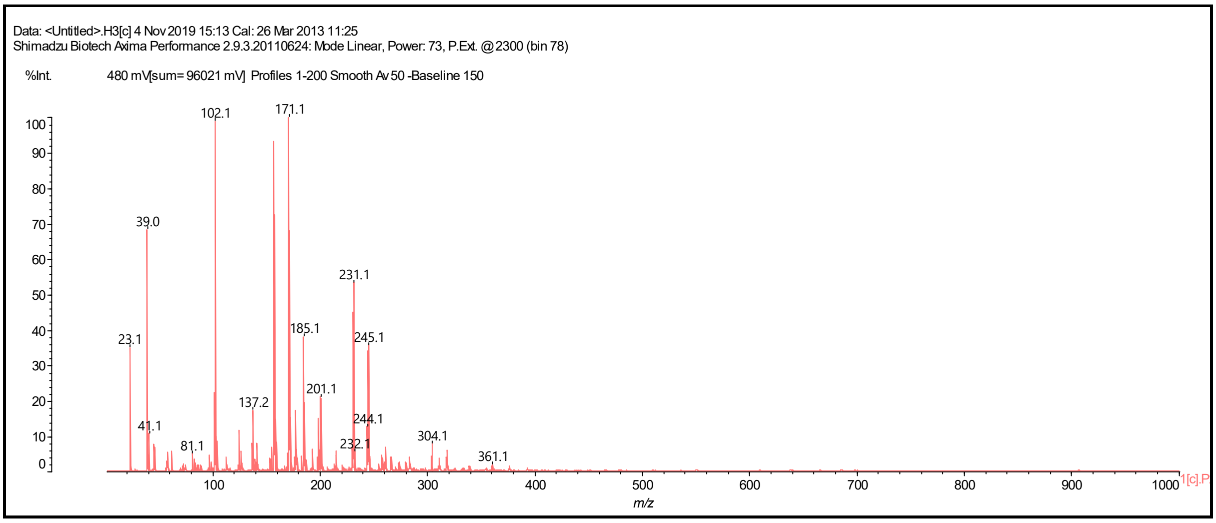

Fig.S1 a

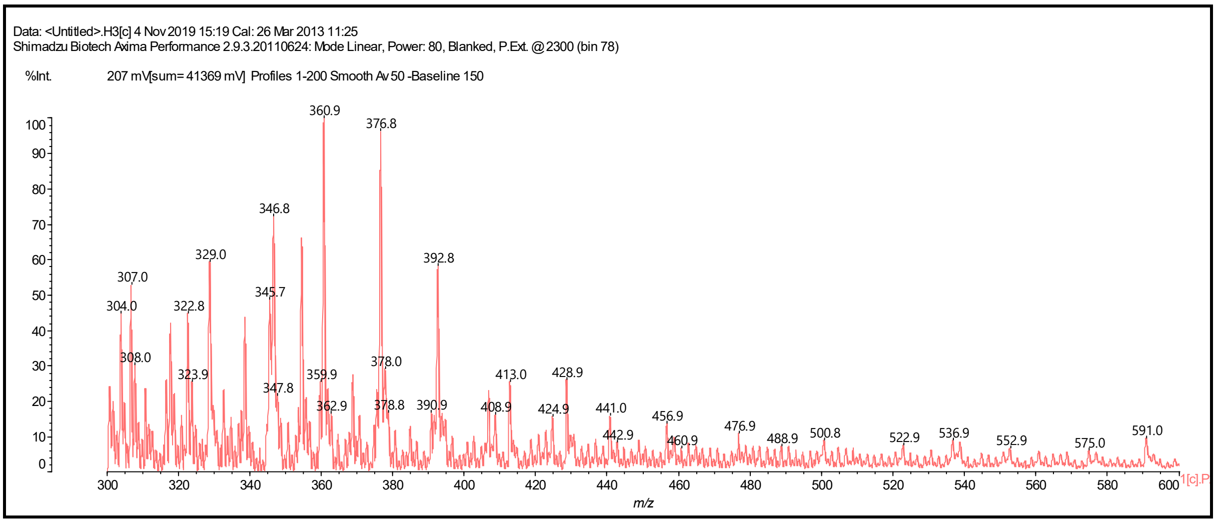

Fig. S1 b

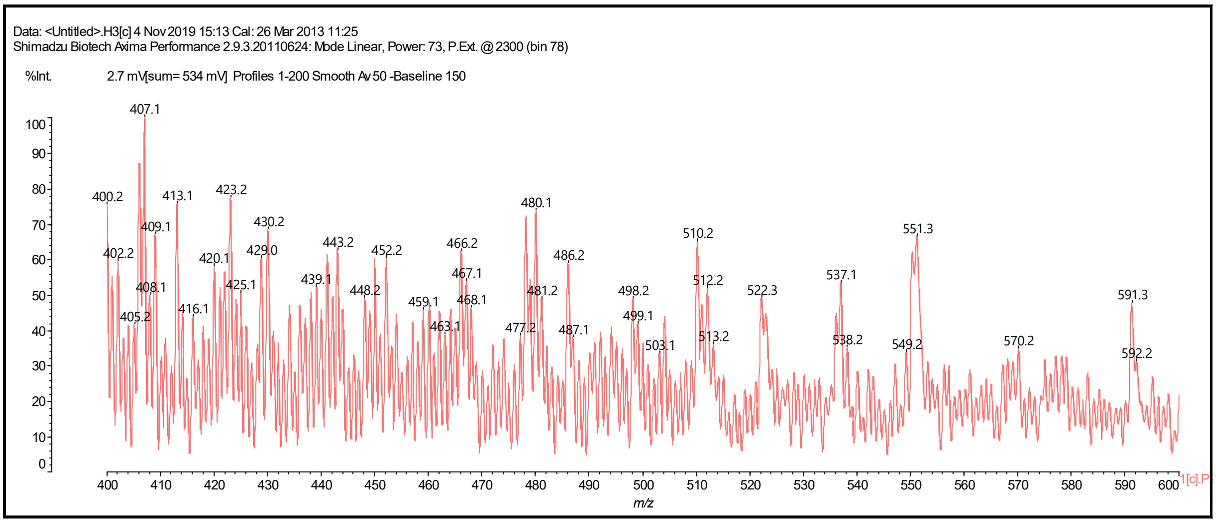

Fig. S1 c

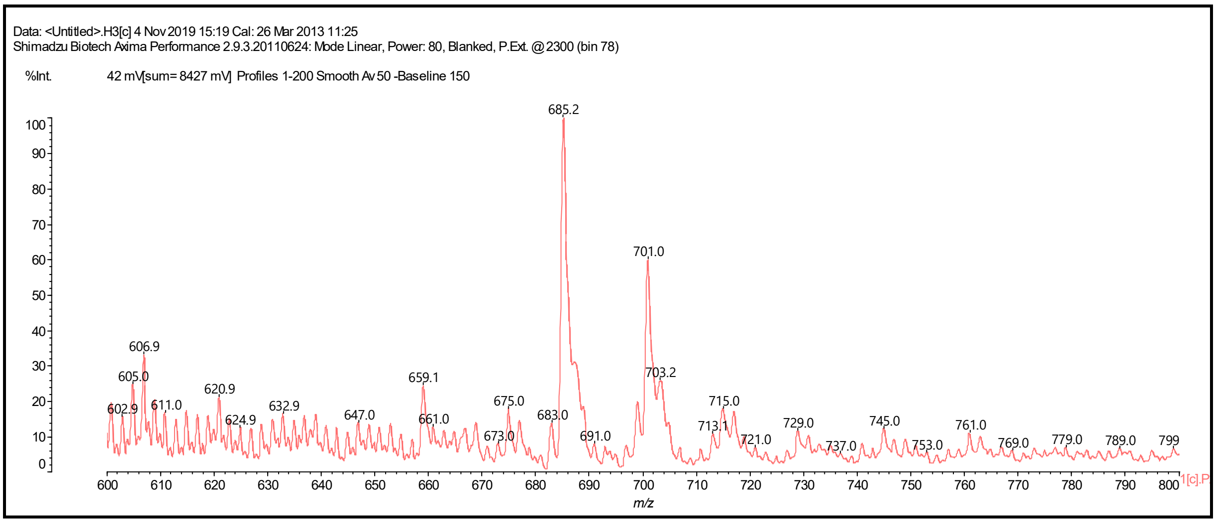

Fig.S1 d

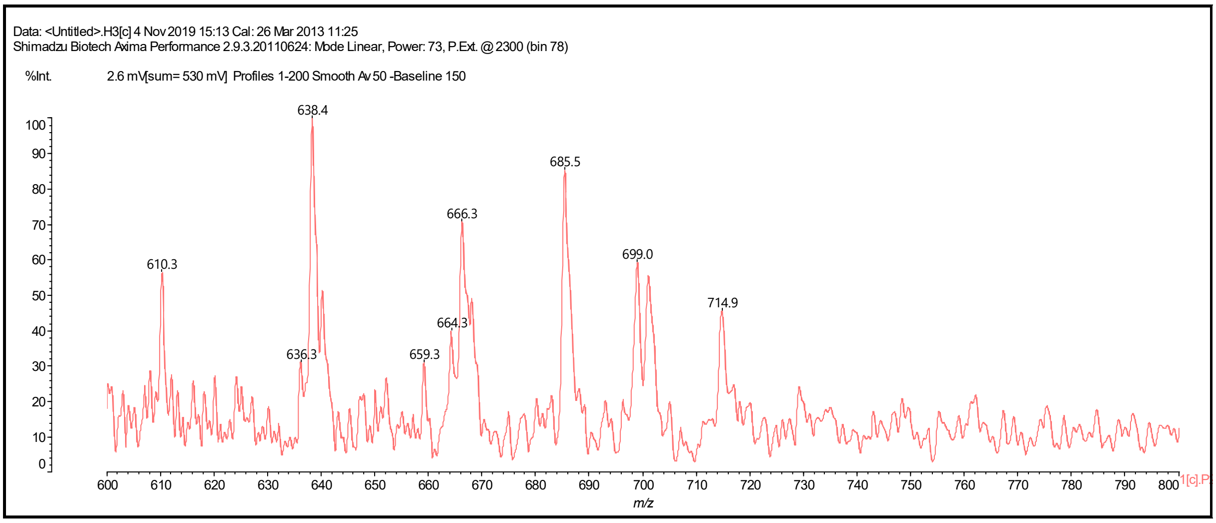

Fig. S1 e

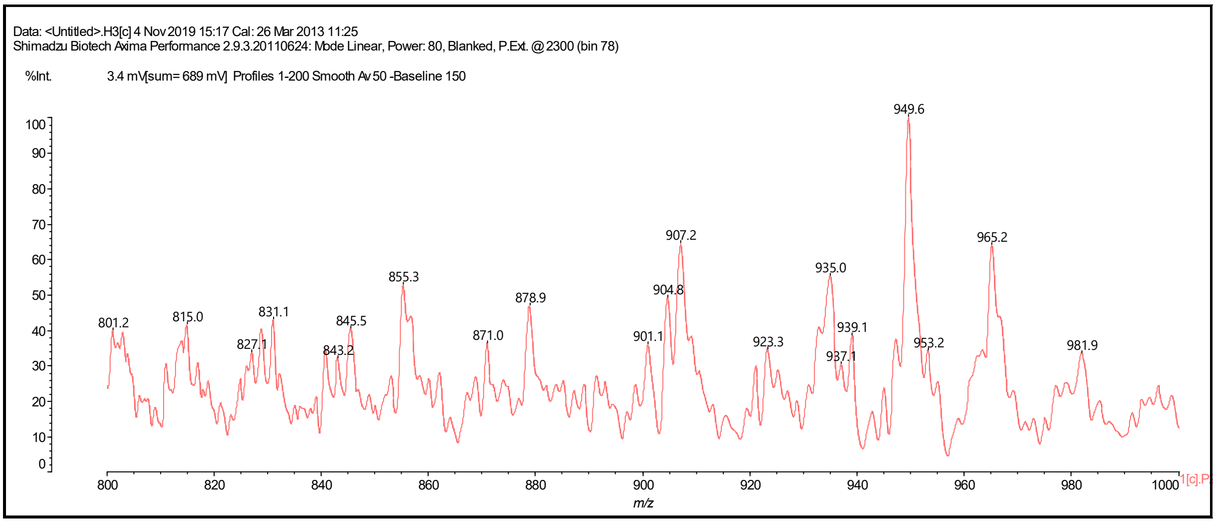

Fig. S1 f

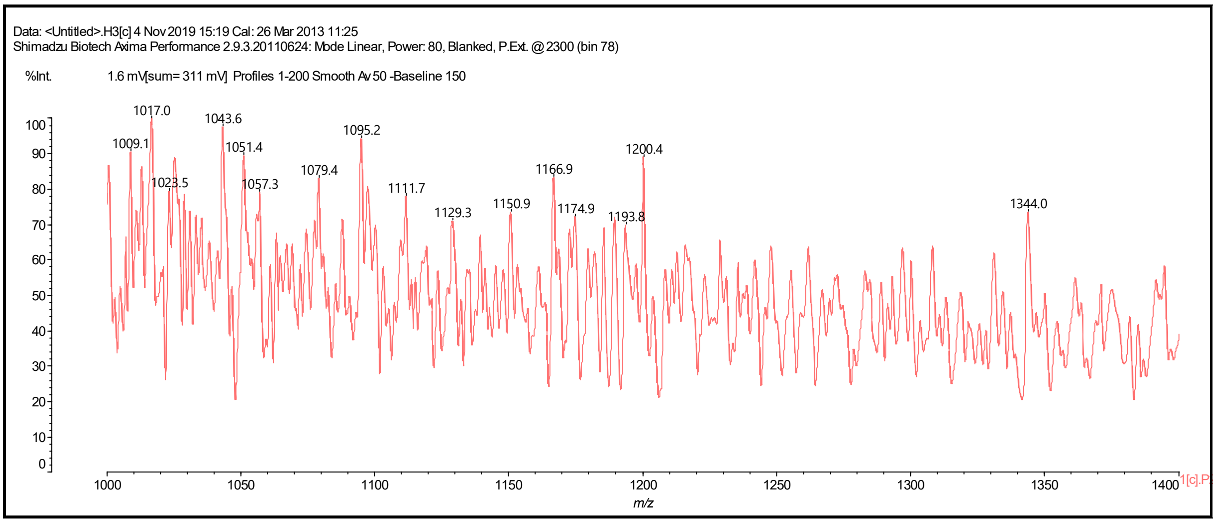

Fig. S1 g

Figure S1. a-g: MALDI ToF spectra details of different Da intervals of raw Gerhumins, corresponding to the structures assigned in Table SM1

Table S2. MALDI ToF assignement of oligomers present in Tannin-Gerhumin NIPU resin A

----

137 Da no Na+

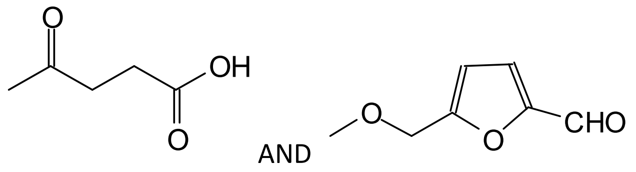

157 Da no Na+

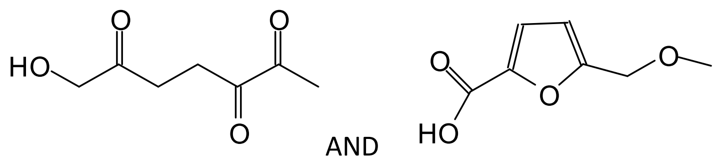

273 Da = Fisetinidin

290 Da = Robinetinidin & Catechin

308 Da = no Na+

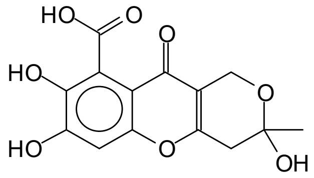

363-364 Da = no Na+ delphinidin+DMC

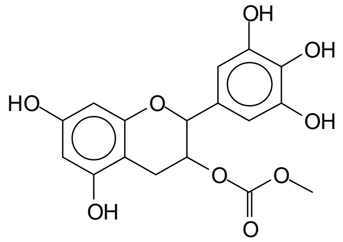

394 Da = with Na+ , deprotonated

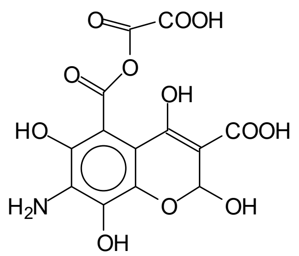

406 Da = no Na+ With the first most probable, but second as reaction with NH_3

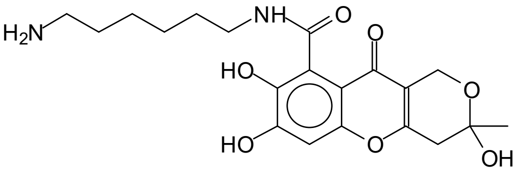

AND

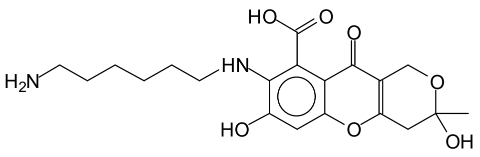

423 Da =406 + 1xOH

465 Da = no Na+, calculated 464 Da

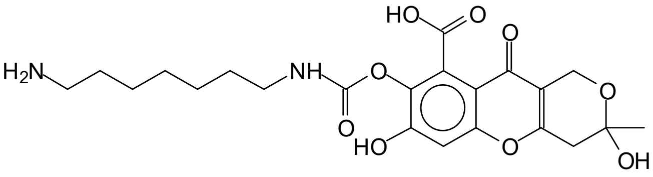

578 Da = no Na+, Robinetinidin or catechin or mixed flavonoid dimer

594-595 Da = no Na+, delphinidin-robinetinidin dimer

621 Da = no Na+, Calculated 622 Da., with Na+ **643-644** Da

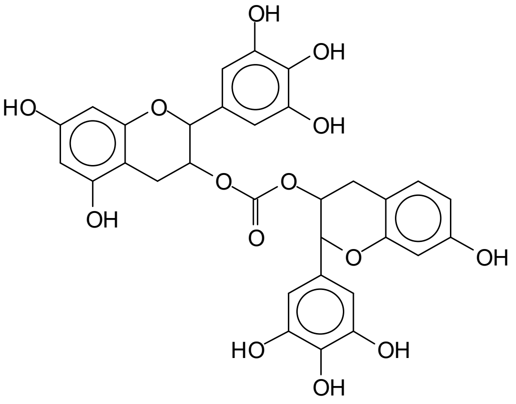

638 Da = no Na+, 1st calculated 638 Da, Second calculated 637 Da

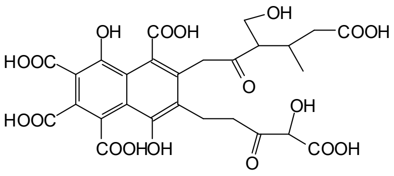

AND/OR

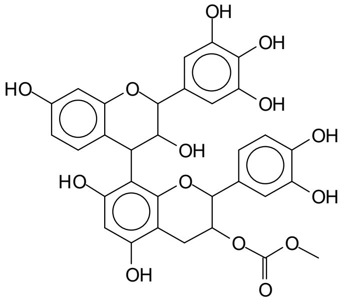

643 Da = with Na+, calculated 645 Da

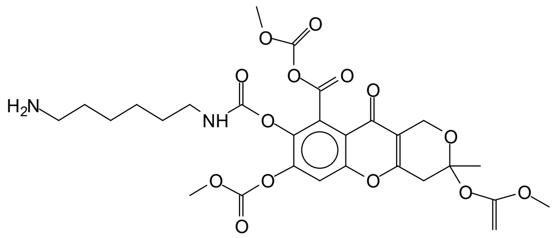

655 Da = 638 Da Nr 2 +1xOH

671 Da = 638 Nr 2 + 2xOHs

698 Da =no Na+, calculated 697 Da

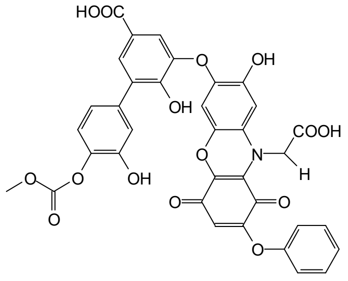

732 Da = no Na+, calculated 732 Da

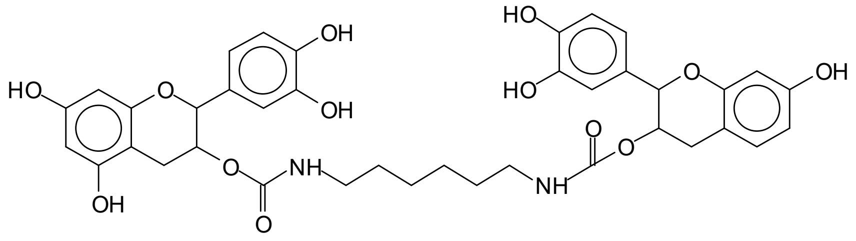

738 Da = no Na+, see also 406 Da note

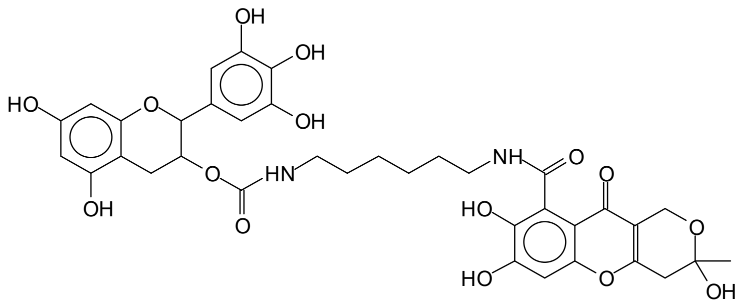

781 Da = no Na+

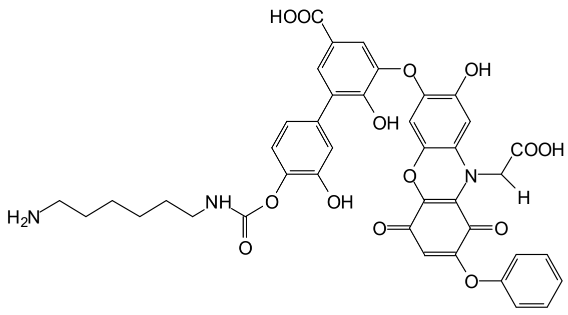

OR

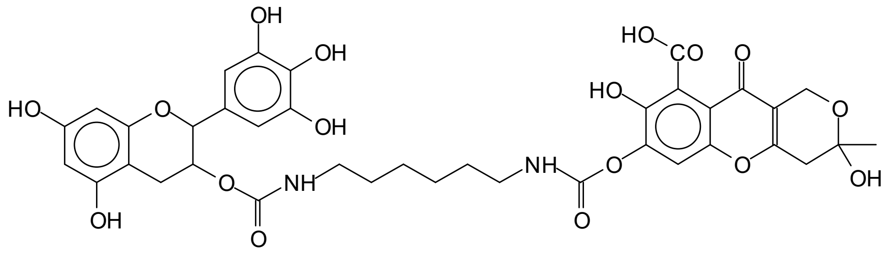

797 Da = no Na+, calculated 796 Da.

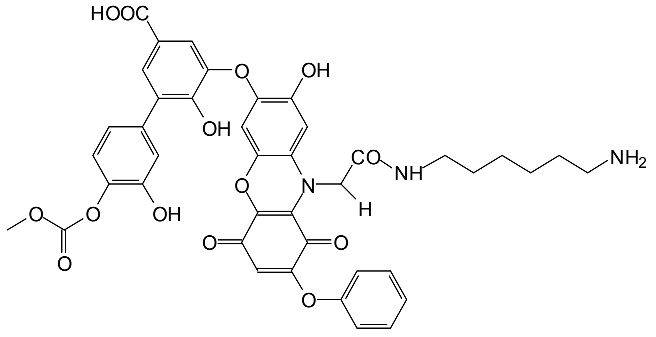

827 Da = nona+

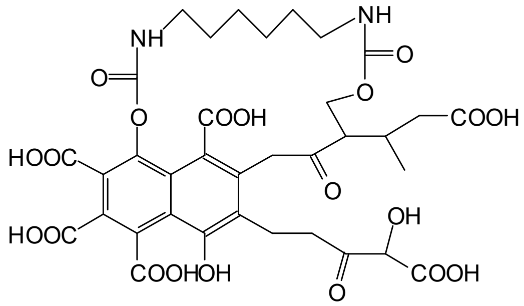

1080 Da =1096 – 1xOH

1096 Da = no Na+, calculated 1096 Da

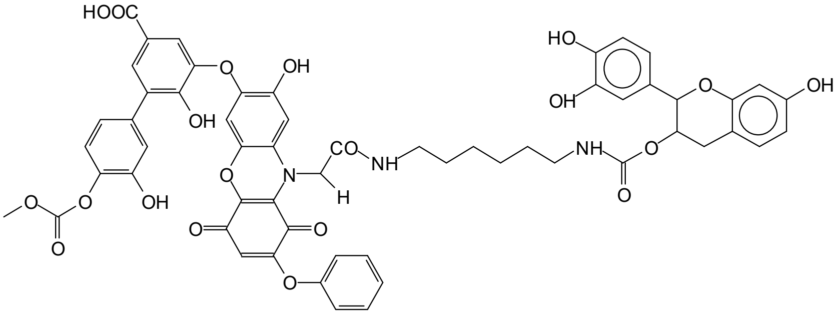

1110 Da = no Na+, calculated 1109.8 Da

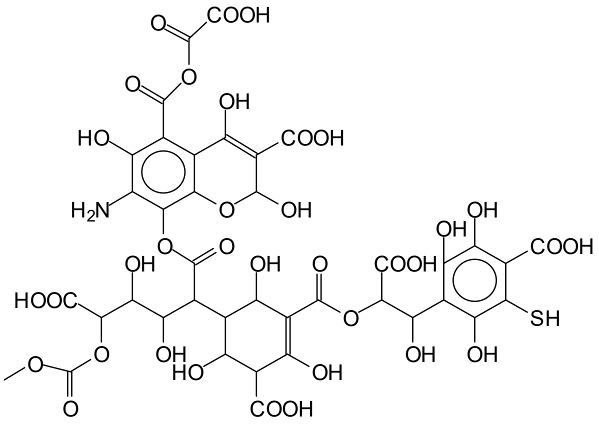

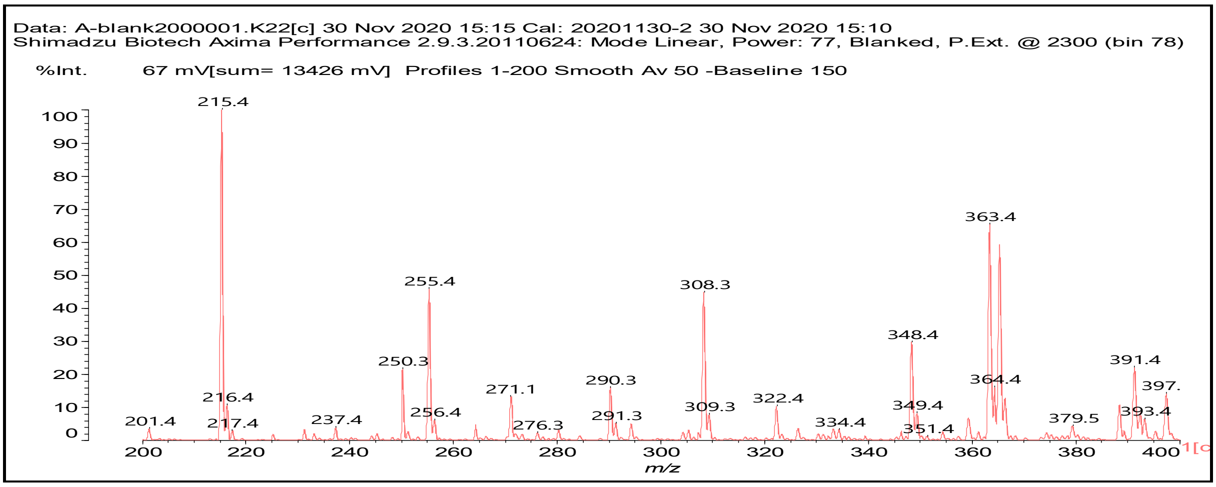

Fig. S2 a

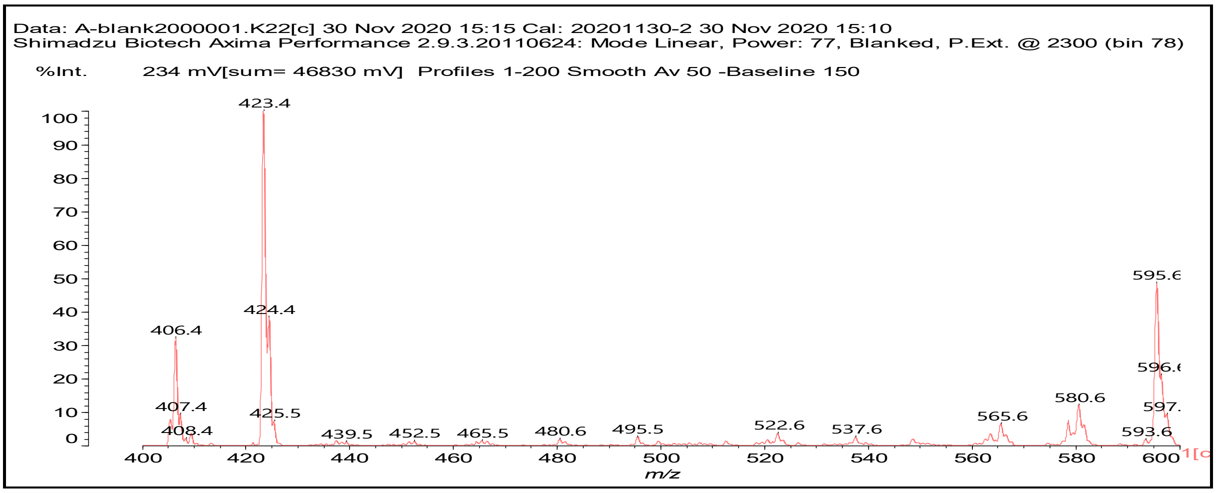

Fig. S2 b

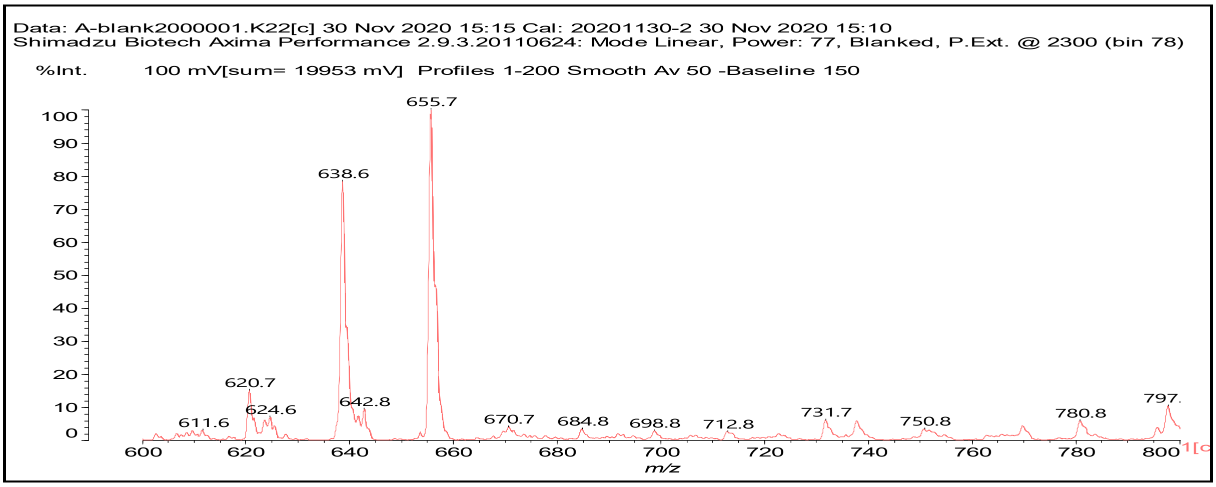

Fig. S2 c

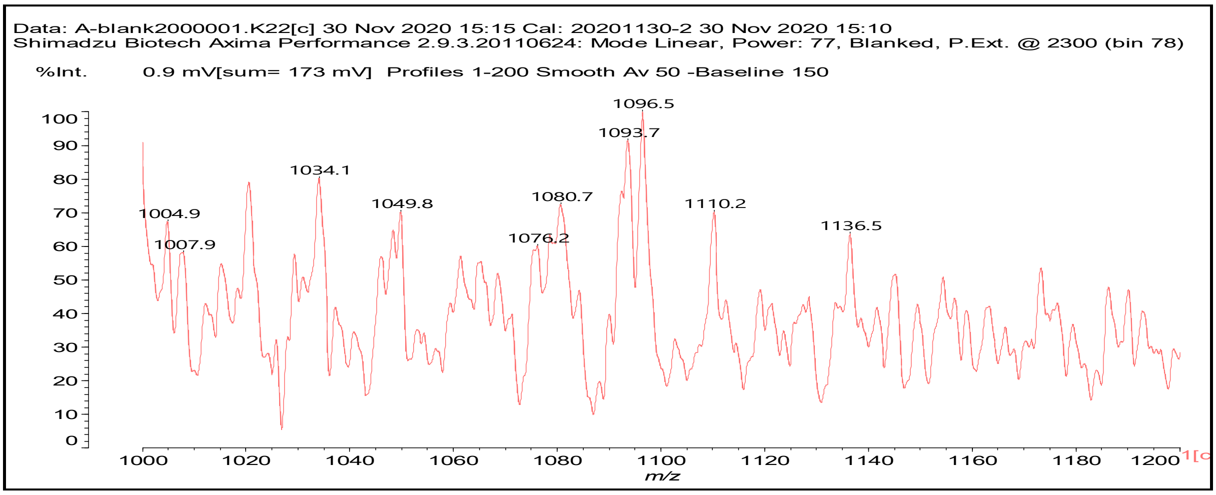

Fig. S2 d

Figure S2. a-d: MALDI ToF spectra details of different Da intervals of Tannin- Gerhumin NIPU Resin A, corresponding to the structures assigned in Table SM2

Table S3. MALDI ToF assignement of oligomers present in pure Gerhumin NIPU resin B

---

250 Da = no Na+

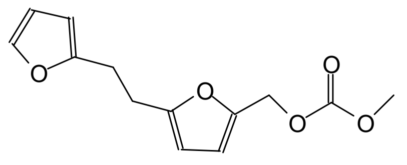

255 Da no Na+ Calculated 254 Da. Furanic Humin unreacted, low

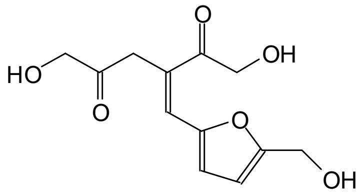

276-277 Da = no Na+, Calculated 280 Da; with Na+

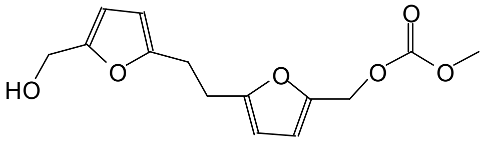

334 Da =no Na+

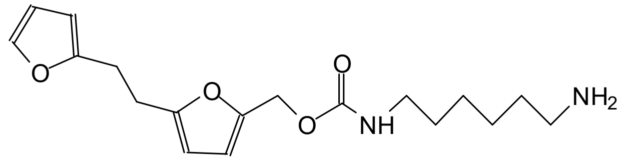

364 Da = no Na+

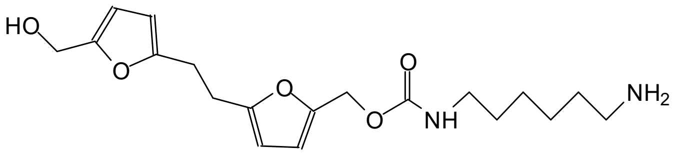

391 Da = no Na+ calculated 389 Da, Furanic Humin unreacted, low

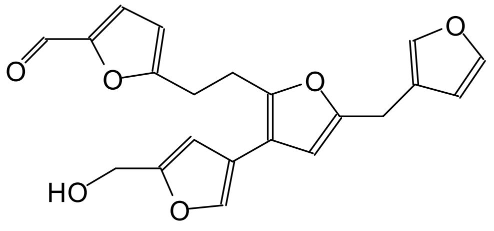

421-423 Da = no Na+, Calcuklated 422 Da

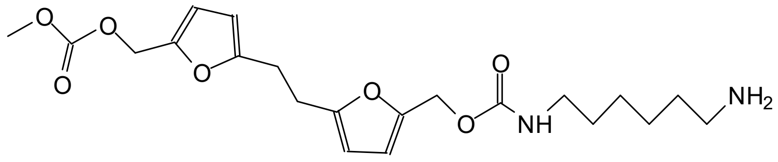

365 Da = no Na+, calculated 366 Da

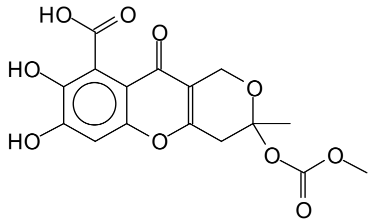

423-424 Da no Na+, calculated 424 Da

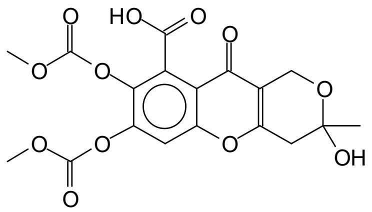

452 Da = no Na+, calculated 451 Da

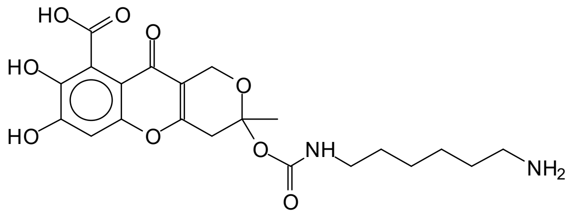

OR, with Na+

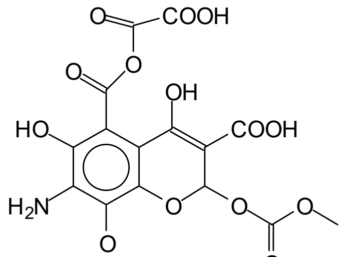

480 Da = no Na+, calculated 480 Da, 3xDMC reacted

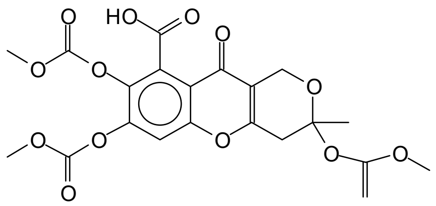

495 Da = noNa+, calculated 494 Da

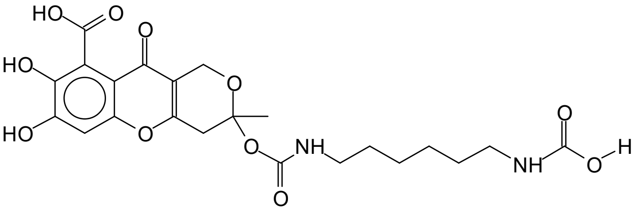

512 Da = no Na+, calculated 512 Da

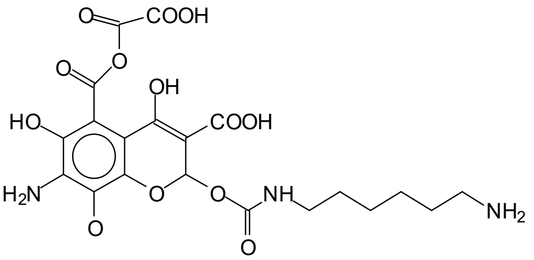

537-538 Da = no Na+, calculated 538 Da Demonstrates that also the -COOH does react with DMC

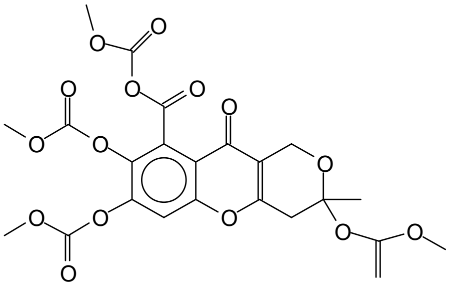

593 Da-595 Da = with Na+

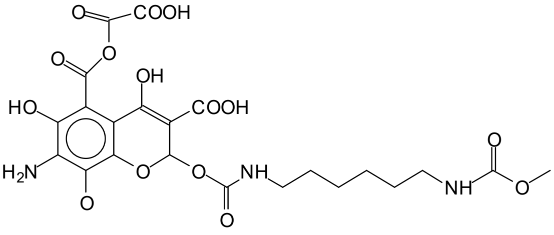

OR

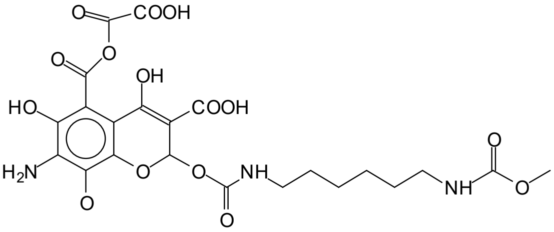

624 Da =no Na+, calculated 623 Da

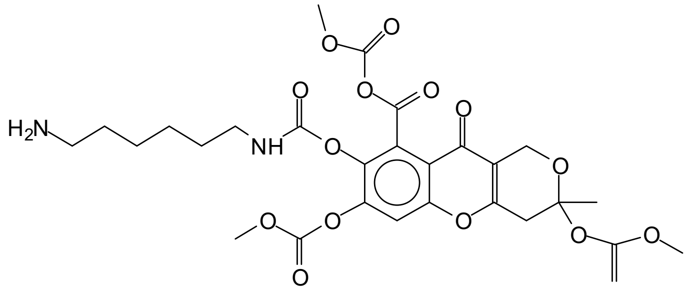

638 Da = with Na+, calculated 637 Da, Furanic Humin unreacted

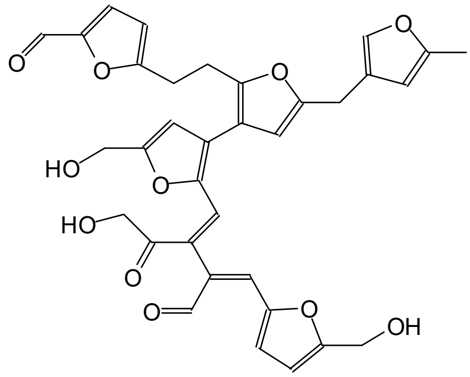

653 Da = with Na+, calculated 651 Da

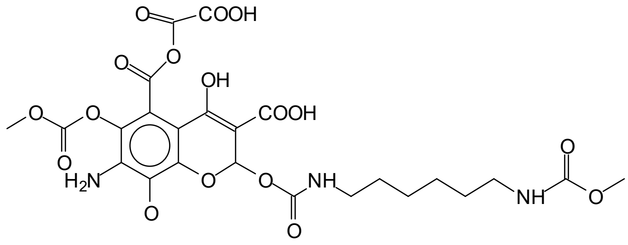

698 Da = no Na+, calculated 697 Da, 721 Da with Na+, calculated 720Da

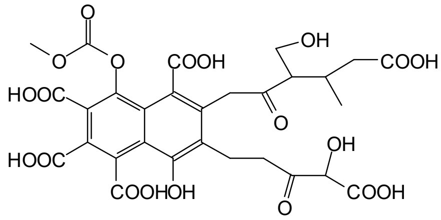

711.5 Da =no Na+, calculated 712 Da, with Na+ = **737 Da** (Calculated 735 Da

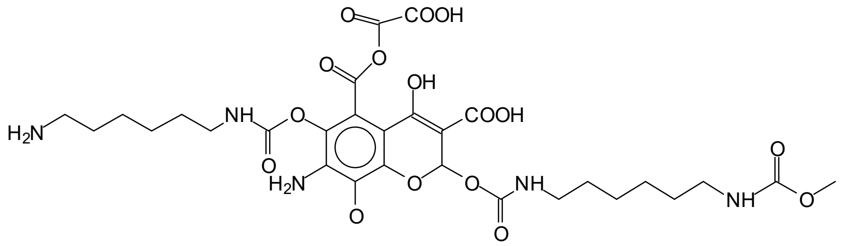

769-770 Da = no Na+, calculated 770 Da

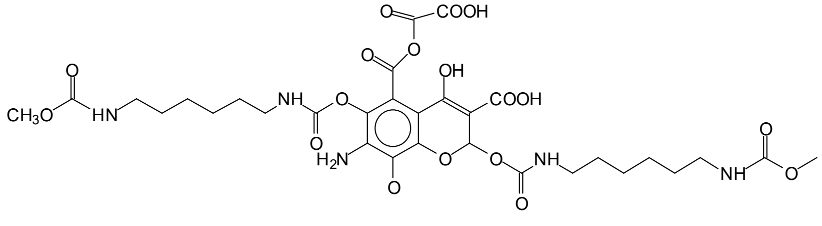

796-798 Da = no Na+, calculated 798 Da 1xCH2 less, 784 Da no Na+, calculated 784 Da (doublé urethane?

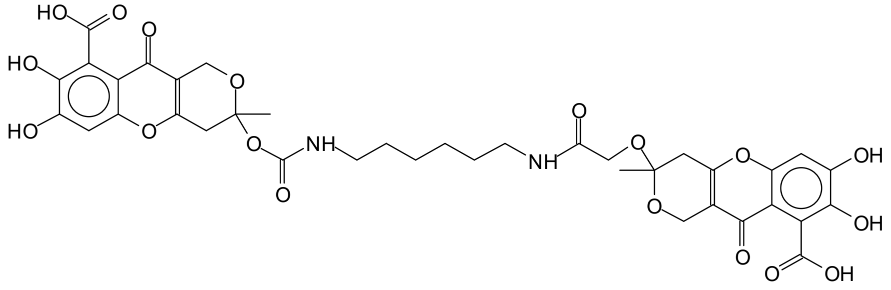

828 Da = with Na+, internal loop of the molecule, calculated 829 Da

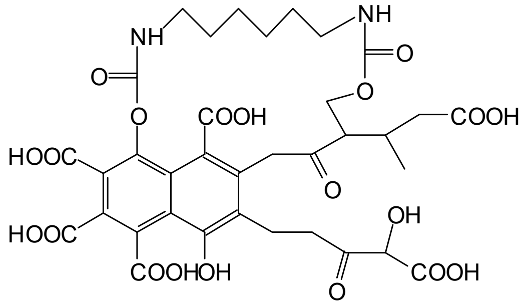

847 Da = no Na+, calculated 847 Da

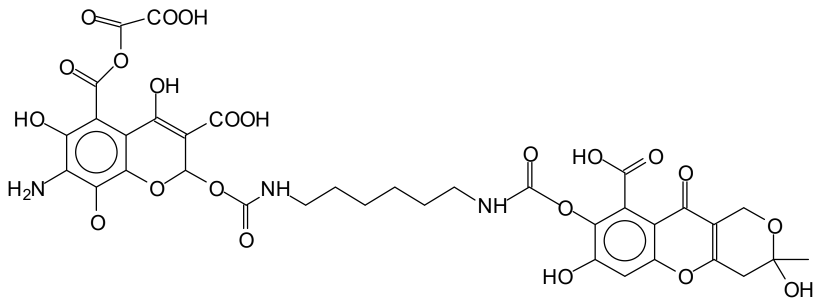

908 Da = no Na+

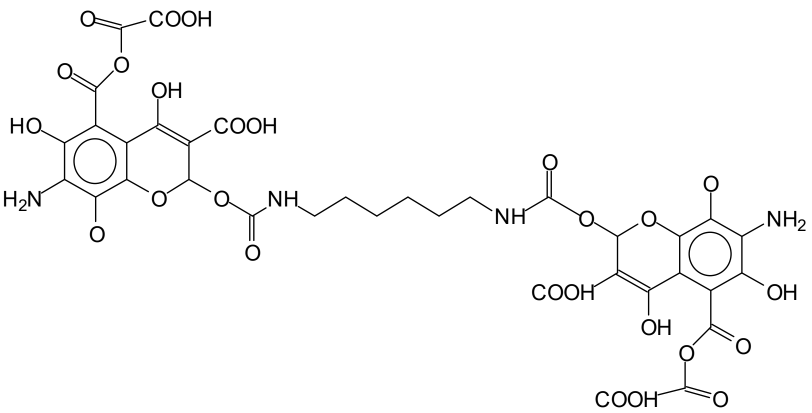

1009 Da = calculated 1012 Da (Experimental furanic Humins (1010Da). No Na+

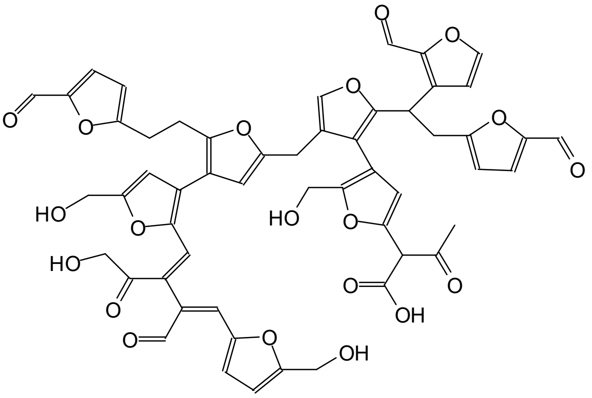

1028 Da = no Na+, calculated 1028 Da a Fulvic acid +DMC+Diamine Linked to a lignan

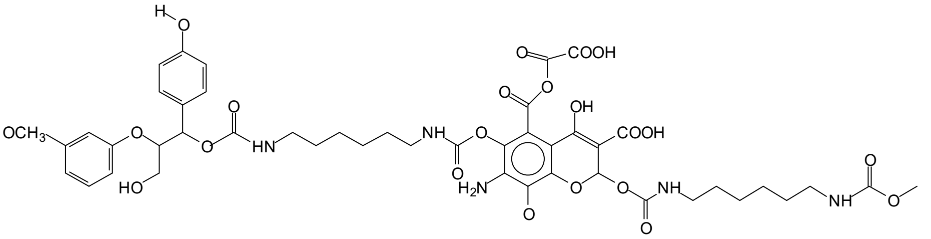

1137 Da (Calculated 1135 Da) no Na+, Furanic Humin fraction unreacted

1193 Da = no Na+, calculated 1194 Da. Reaction with a lignan

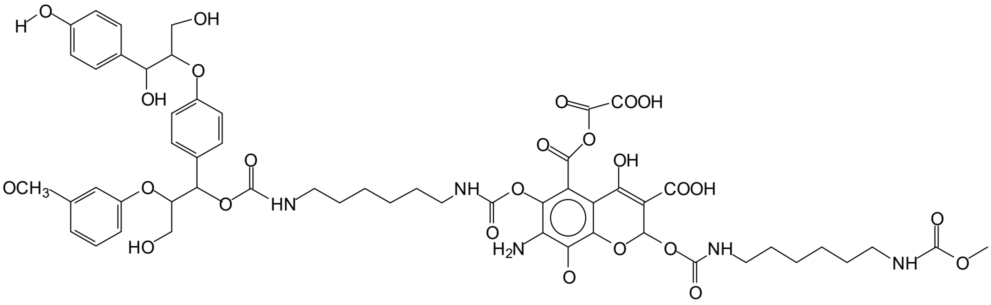

1250 Da (Calculated 1252 Da) with Na+, Furanic Humin fraction unreacted

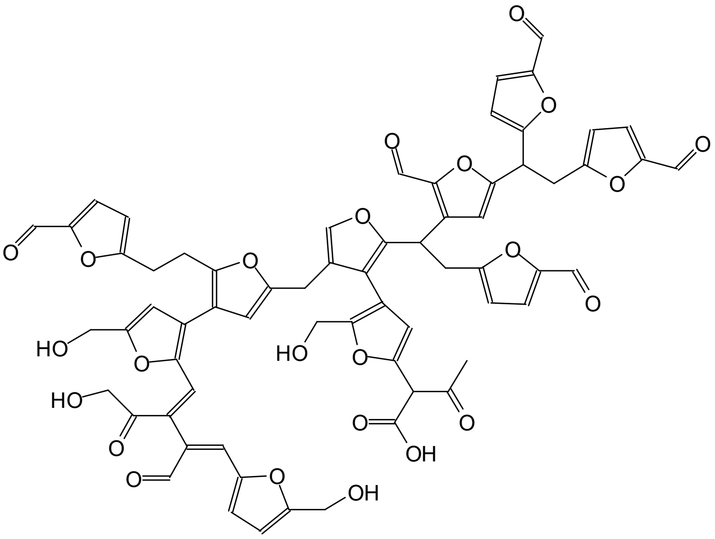

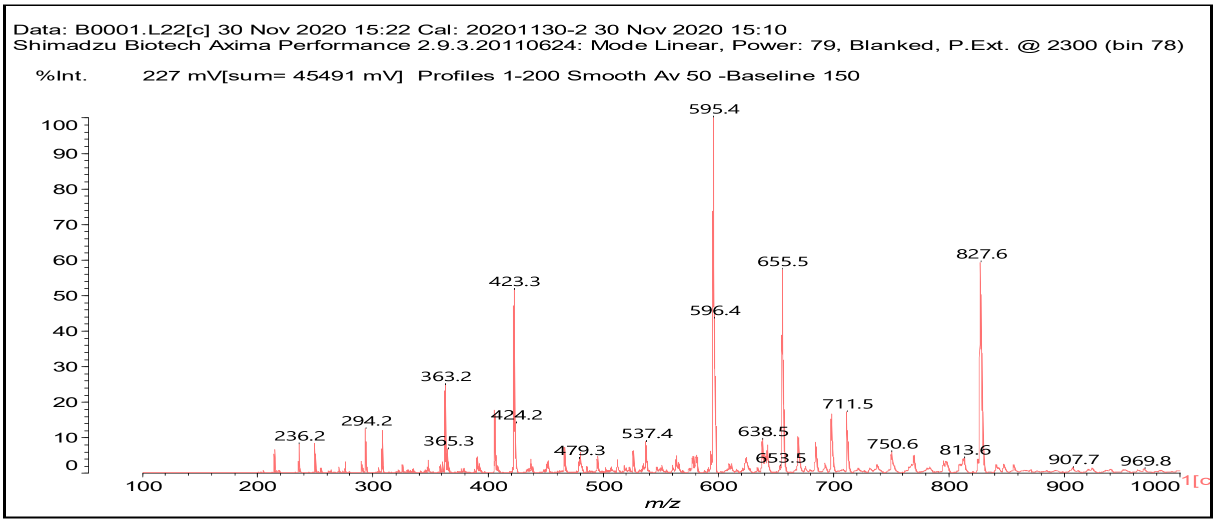

Fig. S3 a

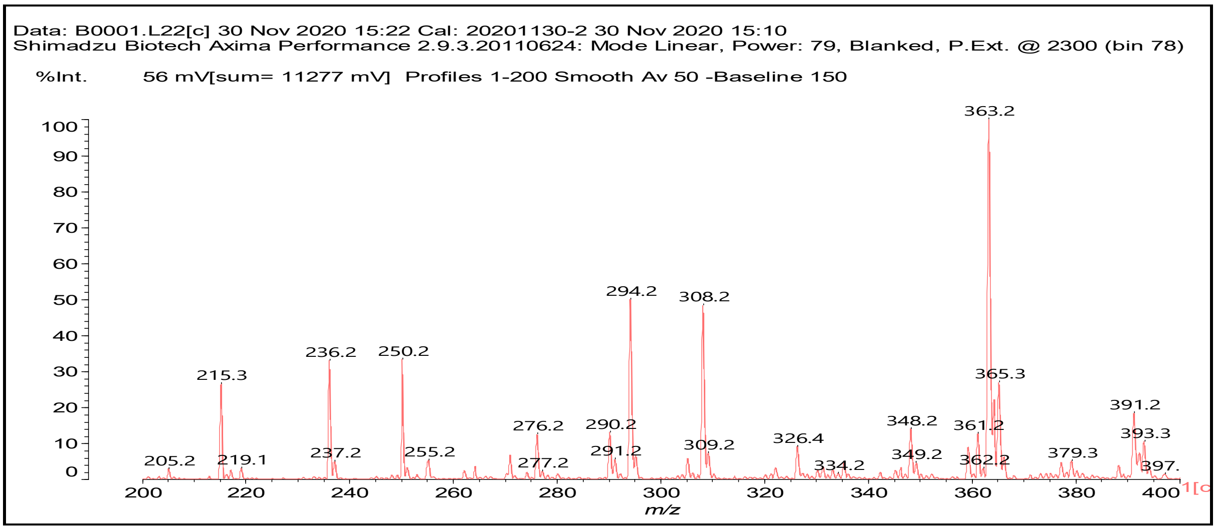

Fig. S3 b

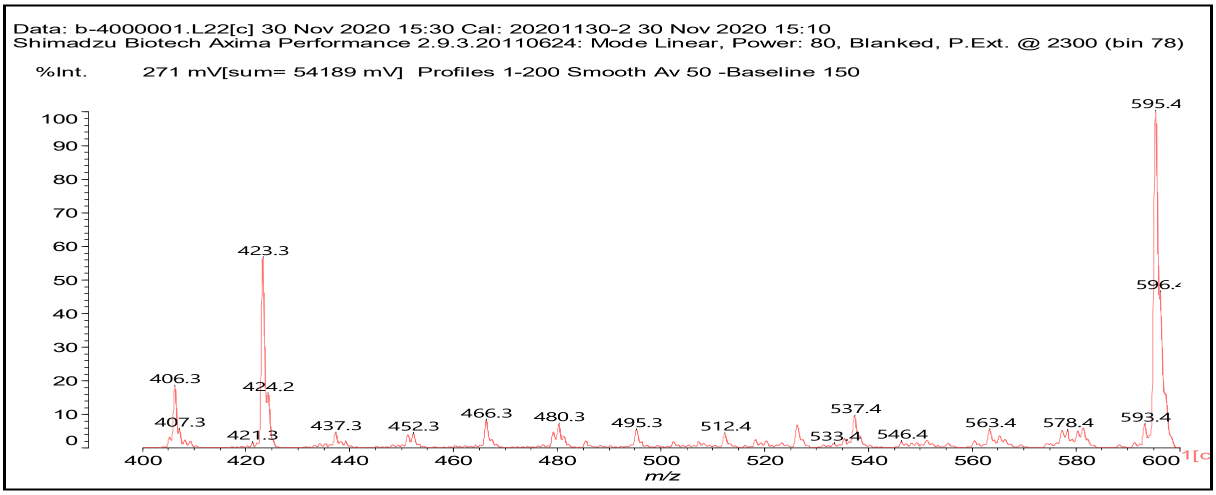

Fig. S3 c

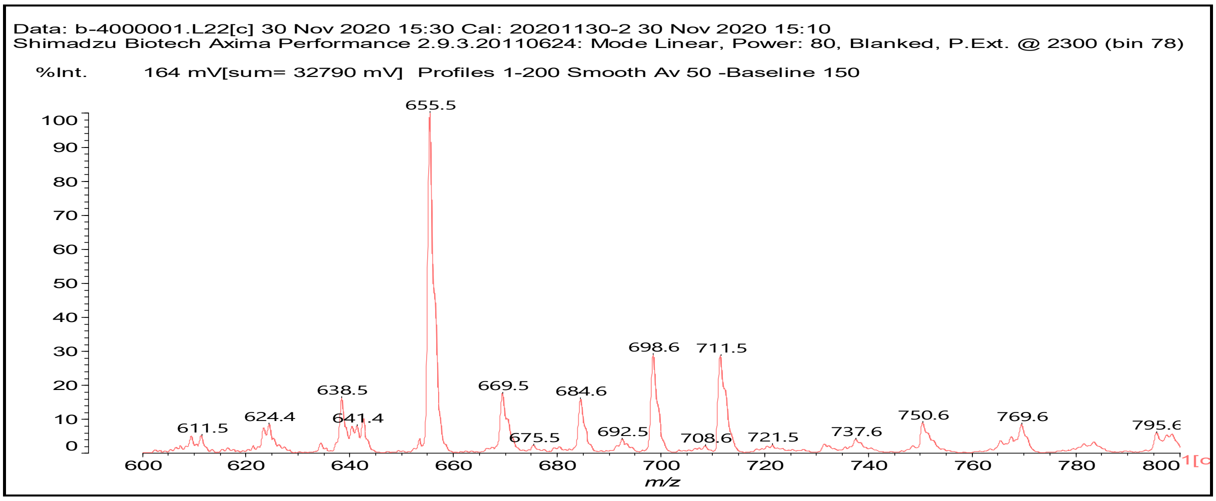

Fig. S3 d

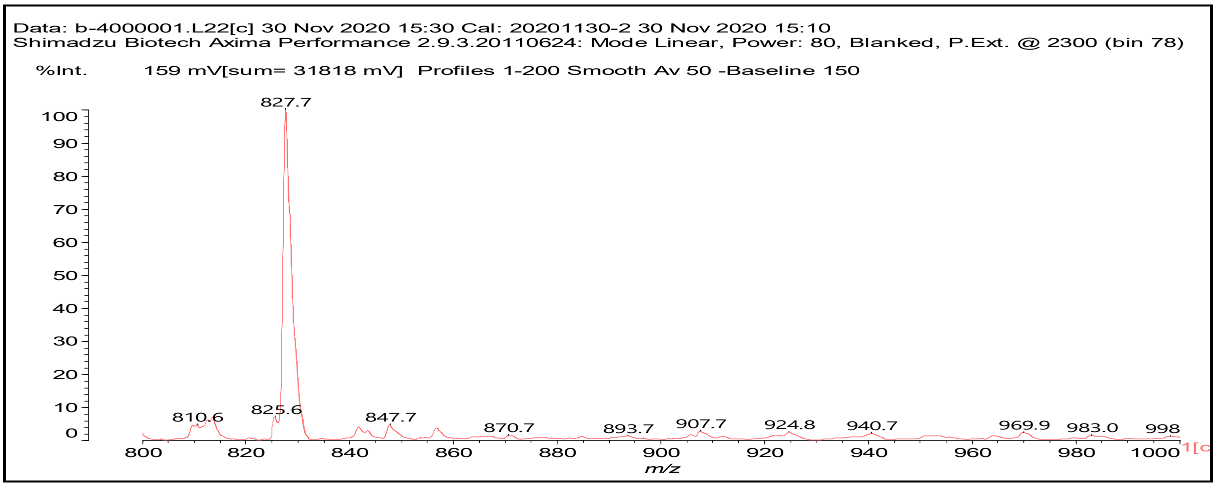

Fig. S3 e

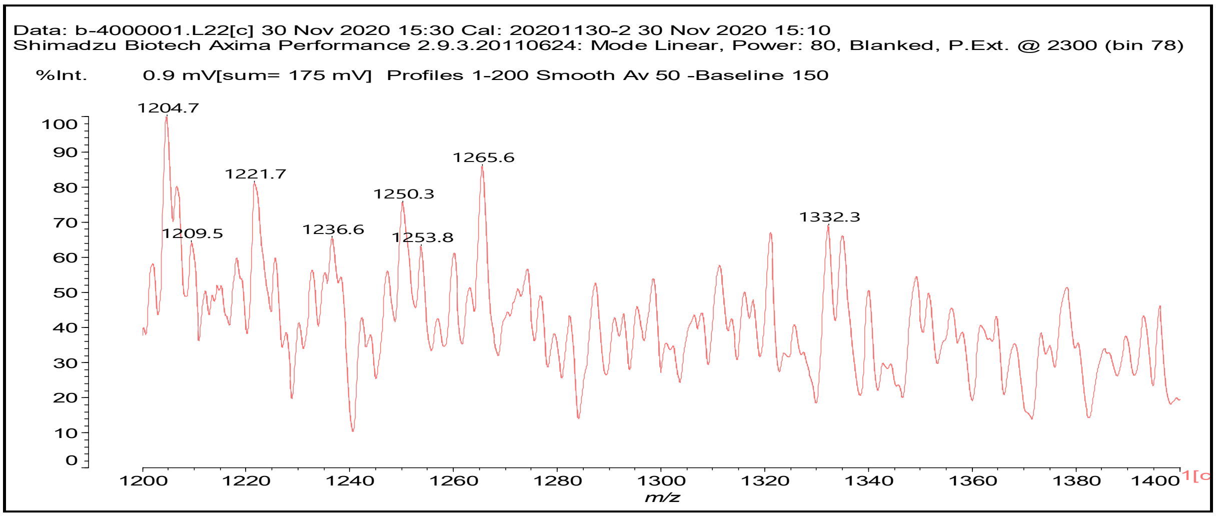

Fig. S3 f

Figure S3. a-f: MALDI ToF spectra details of different Da intervals of pure Gerhumin NIPU Resin B, corresponding to the structures assigned in Table SM3

Table S4. Relative intensity of MALDI peaks for non-furanic humins and tannin-humins NIPU resins.

**A**	**B**	**C**	**D**	
	250			
255		255	255	Furanic humin unreacted
290		290		unreacted flavonoid catechin
	294			
308	308	308	308	fulvic acid unreacted
		326	326	delphinidin+ Na+
363		365		Carbonated delphinidin
348	**348**			
363	363		363	furanic humin urethane
397			397	unreacted fulvic derivate
406	406	406		aminated fulvic acid (no DMC)
423	423	423	423	406 + 1x16
		560	560	robin-fisetin dimer unreacted

578		578	574	mixed flavonoid dimer unreacted
	593			fulvic derivate urethane
595		595	595	unreacted delph-robin dimer
596			595	carbonated fulvic urethane derivate
638	638			carbonated flavon dimer or fulvic derivate
643		642	642	carbonated fulvic monourethane
656	655		655	carbonated fulvic monourethane+1xOH
698	698			carbonated fulvic derivate
	711			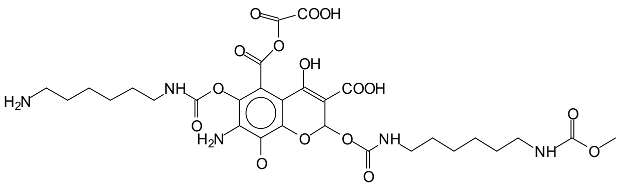
827	827	827	827	Fulvic derivate cyclic diurethane
		885		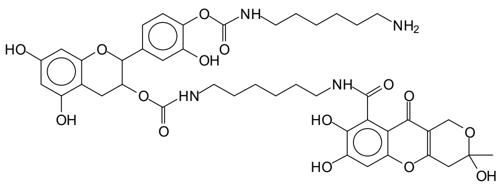
970			970	delphinidin trimer monocarbonated
1095			1093	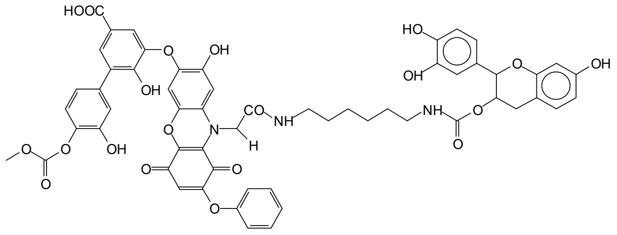
